# Long noncoding RNA repressor of adipogenesis negatively regulates the adipogenic differentiation of mesenchymal stem cells through the hnRNP A1‐PTX3‐ERK axis

**DOI:** 10.1002/ctm2.227

**Published:** 2020-11-08

**Authors:** Yiqian Pan, Zhongyu Xie, Shuizhong Cen, Ming Li, Wenjie Liu, Su'an Tang, Guiwen Ye, Jinteng Li, Guan Zheng, Zhaofeng Li, Wenhui Yu, Peng Wang, Yanfeng Wu, Huiyong Shen

**Affiliations:** ^1^ Department of Orthopedics The Eighth Affiliated Hospital Sun Yat‐sen University Shenzhen China; ^2^ Department of Orthopedics Sun Yat‐sen Memorial Hospital Sun Yat‐sen University Guangzhou China; ^3^ Department of Orthopedics Zhujiang Hospital Southern Medical University Guangzhou China; ^4^ Clinical Research Center Zhujiang Hospital Southern Medical University Guangzhou China; ^5^ Center for Biotherapy Sun Yat‐sen Memorial Hospital Sun Yat‐sen University Guangzhou China

**Keywords:** adipogenesis, long noncoding RNA, mesenchymal stem cell, tissue engineering

## Abstract

**Background:**

Mesenchymal stem cells (MSCs) are pluripotent stem cells that can differentiate via osteogenesis and adipogenesis. The mechanism underlying MSC lineage commitment still remains incompletely elucidated. Understanding the regulatory mechanism of MSC differentiation will help researchers induce MSCs toward specific lineages for clinical use. In this research, we intended to figure out the long noncoding RNA (lncRNA) that plays a central role in MSC fate determination and explore its application value in tissue engineering.

**Methods:**

The expression pattern of lncRNAs during MSC osteogenesis/adipogenesis was detected by microarray and qRT‐PCR. Lentivirus and siRNAs were constructed to regulate the expression of lncRNA repressor of adipogenesis (*ROA*). MSC osteogenesis/adipogenesis was evaluated by western blot and alizarin red/oil red staining. An adipokine array was used to select the paracrine/autocrine factor PTX3, followed by RNA interference or recombinant human protein stimulation to confirm its function. The activation of signaling pathways was also detected by western blot, and a small molecule inhibitor, SCH772984, was used to inhibit the activation of the ERK pathway. The interaction between *ROA* and hnRNP A1 was detected by RNA pull‐down and RIP assays. Luciferase reporter and chromatin immunoprecipitation assays were used to confirm the binding of hnRNP A1 to the *PTX3* promotor. Additionally, an in vivo adipogenesis experiment was conducted to evaluate the regulatory value of *ROA* in tissue engineering.

**Results:**

In this study, we demonstrated that MSC adipogenesis is regulated by lncRNA *ROA* both in vitro and in vivo. Mechanistically, *ROA* inhibits MSC adipogenesis by downregulating the expression of the key autocrine/paracrine factor PTX3 and the downstream ERK pathway. This downregulation was achieved through transcription inhibition by impeding hnRNP A1 from binding to the promoter of *PTX3*.

**Conclusions:**

*ROA* negatively regulates MSC adipogenesis through the hnRNP A1‐PTX3‐ERK axis. *ROA* may be an effective target for modulating MSCs in tissue engineering.

## Graphical abstract

The expression of lncRNA ROA is significantly downregulated during MSC adipogenesis, thereby facilitating the binding of hnRNP A1 to the promoter of PTX3. Increased PTX3 activates the ERK1/2 pathway and promotes the adipogenic differentiation of MSCs in an autocrine/paracrine fashion. By modulating ROA, the efficiency of in vivo MSC adipogenesis can be effectively regulated.

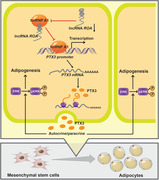

**LIST OF ABBREVIATIONS**
C/EBP‐α: CCAAT/enhancer binding protein alpha
ChIP: chromatin immunoprecipitation
ERK: extracellular signal‐regulated kinase
FABP4: fatty acid binding protein 4
H&E staining: hematoxylin and eosin staining
hnRNP A1: heterogeneous ribonucleoprotein A1
IGFBP2: insulin‐like growth factor binding protein 2
lncRNA *ROA*: long noncoding RNA repressor of adipogenesis
MSC: mesenchymal stem cell
PPAR‐γ: peroxisome proliferator‐activated receptor gamma
PTX3: pentraxin 3
qRT‐PCR: quantitative real‐time polymerase chain reaction
RIP: RNA binding immunoprecipitation

## 1 BACKGROUND

Mesenchymal stem cells (MSCs) are a heterogeneous subset of stem cells with self‐renewal and multilineage differentiation capacities, and osteogenesis, chondrogenesis, and adipogenesis are the three major fates.^1^ Due to their advantages, such as easy accessibility and low immunogenicity, MSCs have drawn great interest from physicians and scientists and hence have been widely applied in tissue regeneration engineering.^2^ These applications rely greatly on the MSC differentiation potential, including osteogenesis and adipogenesis.^3^, ^4^ Dysfunction of their differentiation capacities will not only restrict their clinical use but also lead to the progression of many diseases in vivo.^5^ Therefore, elucidating the mechanism underlying MSC differentiation is of great importance.

Recently, MSCs have been reported to be a major source of adipocytes,^6^, ^7^ and the differentiation of adipocytes has been demonstrated to be linked to diseases, such as obesity, and its comorbidities, such as type 2 diabetes, dyslipidemia, and cardiovascular diseases.^8^ In addition, MSC adipogenesis also becomes promising in tissue engineering and has been utilized for breast augmentation, soft tissue defect filling, and other esthetic or functional purposes.^9^, ^10^, ^11^ However, the regulatory network of MSC adipogenesis has not been fully elucidated and still requires further exploration.

Long noncoding RNAs (lncRNAs) are a type of RNA transcript longer than 200 nt without protein‐coding potential.^12^ They make up a much larger proportion of the human genome than protein‐coding genes^13^ and exert their effects by regulating multiple essential biological events, such as cell proliferation, differentiation, migration, and death.^14^, ^15^, ^16^, ^17^ The differentiation of MSCs is also well regulated by lncRNAs.^18^, ^19^, ^20^ However, although the large estimated number and complex regulatory network of lncRNAs has been reported, knowledge of their roles in MSC lineage commitment remains largely unknown.

In this study, we identified a lncRNA termed repressor of adipogenesis (*ROA*) that negatively regulates the adipogenesis of MSCs. Further mechanistic research revealed that *ROA* functions by preventing hnRNP A1 from binding to the promoter of *PTX3* gene, thus decreasing the expression of the autocrine/paracrine factor PTX3, which then attenuates the activation of the ERK pathway and finally inhibits MSC adipogenesis. Through an in vivo adipogenesis assay, we also demonstrated that by modulating *ROA*, we can effectively regulate the in vivo adipogenic differentiation of MSCs. Our findings provide new knowledge for lncRNA‐regulated MSC adipogenesis and illuminate a possible new way to enhance the efficiency of MSC adipogenic induction in tissue engineering.

## 2 MATERIALS AND METHODS

### 2.1 Stem cell harvest

This study has gained the approval of the ethical committee of The Eighth Affiliated Hospital, Sun Yat‐sen University (Shenzhen, China). Eighteen 20‐ to 30‐year‐old healthy donors were recruited for obtaining MSCs. After explaining the possible risks of bone marrow aspiration, informed consent was signed. Under sterile conditions, bone marrow extraction was performed at the posterior superior iliac spine. The isolation and purification of MSCs were then conducted according to our previously reported methods.^21^ Cells were cultured in flasks with Dulbecco's Modified Eagle's Medium (DMEM; Gibco, Brooklyn, NY, USA) + 10% fetal bovine serum (FBS; Gibco) at 37°C with 5% CO2. Culture medium was replaced at an interval of 2‐3 days. When confluence reached 80‐90%, MSCs were passaged evenly into two flasks by trypsin digestion.

### 2.2 Osteogenic, adipogenic, and chondrogenic differentiation of MSCs

Cells were seeded at a density of 2 × 10^4^ cells/cm^2^ in 12‐well plates for osteogenic and adipogenic induction or in 24‐well plates as high‐density pellets of 6 × 10^5^ cells for chondrogenic induction. The osteogenic medium was made up of DMEM + 10% FBS, 10 mM β‐glycerol phosphate, 50 μM ascorbic acid, and 0.1 μM dexamethasone (all from Sigma‐Aldrich, Darmstadt, Germany). The adipogenic medium was made up of high glucose DMEM + 10% FBS, 0.5 mM 3‐isobutyl‐1‐methylxanthine, 10 μg/mL insulin, 0.2 mM indomethacin, and 1 μM dexamethasone (all from Sigma). The chondrogenic medium was made up of high glucose DMEM + 1% ITS‐Premix (Corning Life Sciences, Madison, WI, USA), 1 mM sodium pyruvate, 0.1 μM dexamethasone, 50 μM ascorbic acid (all from Sigma) + 10 ng/mL rhTGF‐β3 (R&D Systems, Minneapolis, MN, USA). MSCs differentiation was induced with corresponding induction medium. All media were replaced every 2‐3 days until the cells were harvested or fixed for analysis.

### 2.3 Microarray

Total RNA extraction was performed with TRIzol (Thermo Fisher Scientific, Waltham, MA, USA) on days 0 and 10 of MSC osteogenic differentiation. At each timepoint, RNA from three different MSCs was collected and reverse transcribed into cDNA with fluorescent labels for analysis. cDNA was then hybridized with a Long noncoding RNA Microarray v4.0 (Capital Bio Co., Beijing, China). The hybridization signals were detected by a G2565CA microarray scanner (Agilent Technologies, Santa Clara, CA, USA). GeneSpring software (Agilent) was used for data processing and analysis. LncRNAs with a ≥ 2.0 expression fold change and a < .01 *P* value were recognized as significantly differentially expressed. Heat maps and volcano plots were generated using R × 64 3.6.1.

### 2.4 5′‐ and 3′‐ rapid amplification of cloned cDNA ends (RACE)

RACE was conducted using the SMARTer RACE cDNA Amplification Kit (Clontech, Mountain View, CA, USA) as the manufacturer instructed. Briefly, RNA extraction from MSCs was performed using TRIzol (Thermo). First‐strand cDNA was linked to an oligonucleotide adaptor for complete preservation of the 5′‐ and 3′‐cDNA ends. Then, target transcripts were amplified with gene‐specific primers (GSPs) and adaptor primers. The sequences of the GSPs are listed in Table S1. The RACE PCR products were then cloned into a linearized plasmid vector for in‐fusion cloning. Positive clones were picked for sequencing. The sequence of lncRNA *ROA* has been submitted to the GenBank database under accession number MT701605.

### 2.5 In vitro coding ability assay

All plasmid constructs were purchased from OBiO Technology (Shanghai, China). The sequence of *TCONS_0002048* (*ROA*) was artificially synthesized after the start codon and followed by one or two T bases to simulate all possible scenarios for translation. FLAG tags were added to the reconstructed sequences to detect the translation product. pcDNA3.1‐*PKM2*‐FLAG was constructed as a positive control. The reconstructed sequences were cloned into pcDNA3.1(+) plasmid vectors and transfected into 293T cells with Lipofectamine 3000 (Invitrogen). After 72 h, cell lysates were collected and electrophoresed, and a primary antibody against the FLAG tag was used to detect the possible coding product of *ROA*.

### 2.6 Northern blot

DNA probes were labeled with digoxin‐dUTP using the DIG DNA Labeling Kit (Roche, Basel, Switzerland) as the manufacturer instructed. For analysis of *ROA*, whole cell or nuclear/cytoplasmic RNA was separated with a 1‐1.2% agarose gel by electrophoresis in 1 × MOPS solution with 1% formaldehyde and then electro‐transferred onto a Hyoid‐N+ nylon membrane (Beyotime, Shanghai, China). Equilibration of the membranes was done by prehybridization at 65°C for 1 h and then the membranes were hybridized with labeled probes at 65°C overnight. Probe sequences against *ROA* are listed in Table S2. Hybridized probes were detected with Dig Labeled Probe Detection Kit I (Boster, Wuhan, China) as the manufacturer instructed.

### 2.7 Real‐time polymerase chain reaction (qRT‐PCR) and reverse transcription

Cellular RNA was extracted with TRIzol (Thermo). cDNAs were generated through reverse transcription using the PrimeScript RT Reagent Kit (TaKaRa, Dalian, China). A 7500 real‐time PCR detection system (Applied Biosystems, Foster City, CA, USA) was used for detecting qRT‐PCR signals. qPCR system was constructed with harvested cDNAs and SYBR Premix Ex Taq Kit (TaKaRa). The PCR procedure was set as follows: 95°C for 1 min, 42 cycles at 95°C for 30 s + 58°C for 20 s + 72°C for 30 s, and a final 5 min at 72°C for full elongation. Three replicates were performed for each sample and the mean RNA levels were calculated using the 2^−ΔΔCt^ method taking *GAPDH* as the internal control. Specific amplification was evaluated by analyzing the melting curve. The detected genes and their designed primers are listed in Table S3.

### 2.8 Cell cytoplasmic/nuclear fractionation

Cytoplasmic and nuclear fractions were generated using the PARIS Kit (Thermo) as the manufacturer instructed. For each fraction, RNA was isolated and qRT‐PCR was then conducted to evaluate the levels of RNAs of target genes in each fraction. Data were analyzed and presented as the percentages of total RNA. *ACTB* and *GAPDH* were chosen as positive cytoplasmic controls, while *MALAT1* and *U6* served as positive nuclear controls.

### 2.9 RNA interference and transient infection

Small interfering RNAs (siRNAs) for *ROA*, *PTX3*, and *IGFBP2*, as well as a negative control (NC), were generated by GenePharma (Shanghai, China). Sequences of all the siRNAs used in this study are listed in Table S4. MSCs were transfected with specific siRNAs or NC siRNAs at a dose of 1 OD per 1.5 × 10^6^ cells with Lipofectamine RNAiMAX (Thermo). The transfection medium was removed after 6 h and MSCs were harvested for analysis of the knockdown efficiency by qRT‐PCR 72 h after RNAi.

### 2.10 Lentivirus construction and infection

The *ROA* and hnRNP A1 overexpression lentivirus and NC were ordered from OBiO Technology. MSCs were infected with the overexpression Lentivirus or NC Lentivirus (MOI = 50) together with 5 μg/mL polybrene in the transfection medium for 24 h and then the Lentivirus‐containing mixture was removed. After 72 h, the cells were harvested for analysis of overexpression efficiency by qRT‐PCR.

### 2.11 Western blot

RIPA lysis buffer (Sigma) containing 1% protease and phosphatase inhibitors (Thermo) was added to prewashed cells for obtaining cell lysates. The lysis step was conducted on ice for 30 min. Cell lysates were then collected and centrifuged at 4°C at the rotate speed of 12 000 rpm for 30 min. The supernatant was collected and sent for protein quantification using a BCA assay kit (Thermo). 10% SDS‐polyacrylamide gel electrophoresis was then conducted to separate proteins of different molecular weights. The separated proteins were then electro‐transferred onto polyvinylidene fluoride membranes (EMD Millipore, Burlington, MA, USA). Membrane blockage was performed with 5% skim milk at room temperature for 60 min. Then, incubation was performed overnight at 4°C with primary antibodies against GAPDH, RUNX2, OCN, PPAR‐γ, FABP4, C/EBP‐α, SOX9, IGFBP2, PTX3, β‐catenin, N‐p‐β‐catenin, ERK1/2, pERK1/2, AKT, pAKT, JNK, pJNK, and hnRNP A1. Detailed information for the primary antibodies used for western blot is all listed in Table S5. Thereafter, nonspecific binding was removed with TBST washes and the membranes were incubated with HRP‐conjugated secondary antibodies (diluted 1:2000; Santa Cruz Biotechnology, Santa Cruz, CA, USA) at room temperature for 60 min. Unbound antibodies were washed away with TBST. The luminous substrate was generated using the Immobilon Western Chemiluminescent HRP Substrate (Millipore) and an imaging system was used to detect signals. Densitometry was performed using ImageJ software (National Institutes of Health, Bethesda, MD, USA). The intensity of GAPDH of each sample was used for normalization. Parallel gels and stripping and reprobing methods were used to detect proteins of similar molecular weight.

### 2.12 Alkaline phosphatase activity and staining

After 7 days of osteogenic induction, MSCs were harvested for alkaline phosphatase (ALP) activity measurement or fixed for ALP staining. ALP activity was assessed with an ALP assay kit (Nanjing Jiancheng Bioengineering, Nanjing, China) as the manufacturer instructed. Total protein was quantified using a BCA assay kit (Thermo). ALP activity was presented as catalytic units per gram of protein per 15 min. ALP staining was performed with a BCIP/NBT Alkaline Phosphatase Color Development Kit (Beyotime) as the manufacturer instructed.

### 2.13 Alizarin red S and oil red O staining and quantification

After 14 days of osteogenic induction or 12 days of adipogenic induction, MSCs were fixed with 4% paraformaldehyde for staining. The alizarin red S (ARS) dye was made up of 1% ARS (pH 4.3) and the oil red O (ORO) dye was made up of 0.3% ORO dissoleved in 60% isopropyl alcohol. A 20‐min staining step was conducted at room temperature. Thereafter, the dye was removed, and nonspecific staining was washed away with PBS. Then, the stained cells were observed and photographed under a microscope. 10% cetylpyridinium chloride monohydrate (Sigma) was used to extract combined ARS and isopropyl alcohol was used to extract combined ORO. Then, a 200‐μL aliquot was transferred to a 96‐well plate for measurement of absorbance at 562 nm for ARS and 520 nm for ORO.

### 2.14 Cytokine array assay

MSCs were cultured in 12‐well plates at a density of 2 × 10^4^ cells/cm^2^. After 3 days of *ROA* knockdown, culture supernatants were collected from each group and analyzed using a Human Adipokine Array Kit (R&D Systems) as instructed. Each assay was performed with 500 μL of culture supernatant. The pixel density of each spot was measured by ImageJ (NIH). Mean pixel densities were normalized to the reference spots.

### 2.15 Exogenous PTX3 and IGFBP2 assay

Recombinant human PTX3 (Abcam) was added to the adipogenic medium at concentrations of 0, 100, and 200 ng/mL, and recombinant human IGFBP2 (Abcam) was added at concentrations of 0, 50, and 100 ng/mL for stimulation. The culture medium was replaced every 2‐3 days until MSCs were harvested or fixed for other assays.

### 2.16 RNA pull‐down and mass spectrometry assays

The TranscriptAid T7 High Yield Transcription Kit (Thermo) was used for in vitro transcription of ROA, its fragmented sequences, and antisense transcript. The RNA products were then purified and labeled with biotin using the Pierce 3′ End Biotinylation Kit (Thermo). RNA pull‐down was performed using the Pierce™ Magnetic RNA‐Protein Pull‐Down Kit (Thermo). Briefly, biotin‐labeled RNAs were incubated with cell lysates in the binding buffer for 2 h and collected by magnetic beads. The pull‐down proteins were then eluted and underwent SDS‐PAGE for separation. Silver staining was conducted to detect differentially pulled protein bands. Protein bands differentially enriched by *ROA* and its antisense transcript were collected for mass spectrometry.

### 2.17 RNA‐binding immunoprecipitation

The Magna RIP Kit (Millipore) was used for the RNA‐binding immunoprecipitation (RIP) assay as instructed. MSCs were first treated with RIP lysis buffer. The proteins were then harvested for incubation with magnetic beads conjugated to anti‐hnRNP A1 antibody or rabbit IgG NC. The precipitated RNAs were then purified, and the target RNA was amplified by PCR for detection.

### 2.18 Dual‐luciferase reporter assay

The promoter sequence of *PTX3* was synthesized and cloned into pGL4.10 plasmid vectors, and transfected into 293T cells with Lipofectamine 3000 transfection reagent (Invitrogen). Sequences of *hnRNP A1* and *ROA* were also synthesized and cloned into pcDNA3.1(+) plasmid vectors and transfected into 293T cells in corresponding wells for overexpression. Internal control was set by cotransfacting pRL plasmids. Luciferase activities among different groups were detected by the Dual‐Luciferase Reporter Assay System (Promega, Fitchburg, WI, USA). Relative luciferase activities were calculated as the ratio of firefly/Renilla luciferase activity.

### 2.19 Chromatin immunoprecipitation assay

The EZ‐Magna ChIP A/G Assay Kit (Millipore) was used for chromatin immunoprecipitation (ChIP) assays according to the instruction manual from the manufacturer. Cells were treated with 1% formaldehyde for chromatin crosslinking and then collected and lysed with cell membrane extraction buffer to obtain the nuclei. Chromatin was then sheared by sonication into 200‐1000 bp DNA fragments and immunoprecipitated with normal rabbit IgG control or an anti‐hnRNP A1 antibody (Abcam). The precipitated DNA was then purified and went through PCR amplification for detection. ChIP primers designed for qPCR are listed in Table S6.

### 2.20 In vivo adipogenesis assay

This experiment has gained the approval of the Animal Ethical and Welfare Committee of The Eighth Affiliated Hospital, Sun Yat‐sen University (Shenzhen, China). The experiment was conducted as previously reported with minor modifications.^22^ MSCs at the fourth passage were modified in vitro either by lncRNA *ROA* overexpression or knockdown and underwent adipogenic induction for 5 days before the grafting assay. On day 6, cells were trypsinized, counted, and resuspended in Matrigel (BD Biosciences, San Jose, CA, USA). Before the operation, 8‐week‐old male nude mice (Gempharmatech, Jiangsu, China) were anesthetized with 10 mL/kg 4% chloral hydrate. Matrigel (1.5 × 10^4^ cells/150 μL) was then grafted subcutaneously into the backs of nude mice (n = 4 per group) by injection at symmetrical sites. After 8 weeks, MSC/Matrigel plugs were taken out, fixed with 4% paraformaldehyde, embedded in paraffin, and sectioned for H&E staining or immunohistochemistry. Quantification was done by calculating the percentage of fat area and fat vacuole number per mm^2^ with ImageJ software.

### 2.21 H&E staining and immunohistochemistry

Sections of MSC/Matrigel plugs were generated, followed by deparaffinization and hydration. For H&E staining, the dying procedure was 10 min of hematoxylin staining, clearance in 70% alcohol containing 1% HCl, and 4 min of eosin staining. For immunohistochemistry, a primary antibody against human perilipin‐1 (diluted 1:200; Abcam) was used for staining. An SP Rabbit & Mouse HRP Kit (DAB) (Cwbio, Beijing, China) was used for color development. The stained sections were visualized and photographed under a light microscope (Nikon, Japan).

### 2.22 Statistical analyses

SPSS 24.0 (IBM, Chicago, IL, USA) was used for analyzing all the statistics. Data are presented as the means ± SDs. Statistical difference was determined by the Student's *t*‐test (between two groups) and one‐way analysis of variance (ANOVA) followed by Bonferroni's test (among three or more groups). For correlation analysis, the Pearson correlation test was conducted. *P* value less than .05 was considered statistically significant.

## 3 RESULTS

### 3.1 *ROA* negatively regulates the adipogenesis of MSCs rather than osteogenesis

**FIGURE 1:**
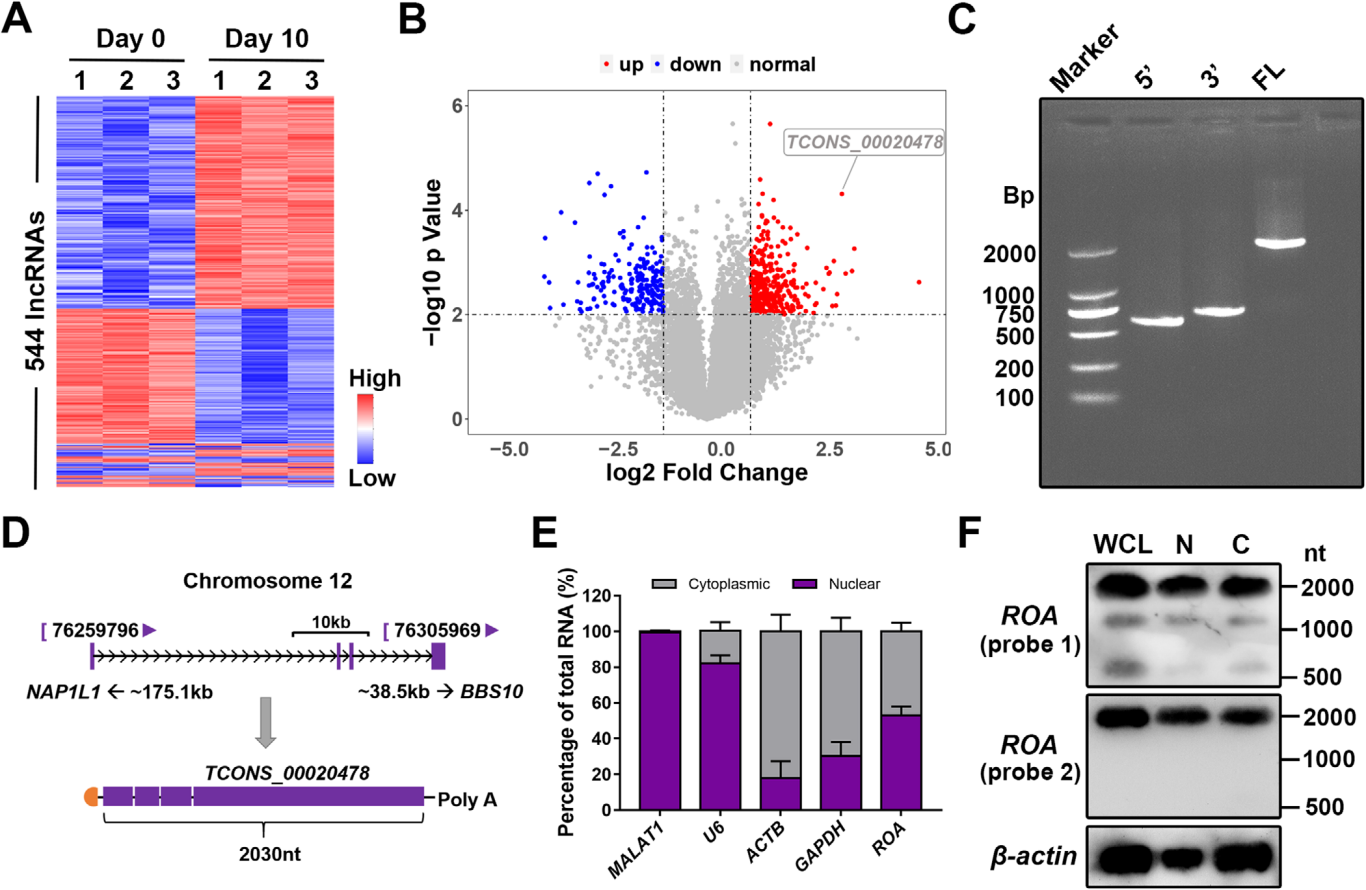
***TCONS_00020478* was selected by microarray**. A, Cluster heatmap showing lncRNAs with an expression fold change ≥ 2 from microarray data on days 0 and 10 of MSC osteogenesis (n = 3, *P* < .01). B, Volcano plot of 20 703 analyzed lncRNAs. *ROA* (*TCONS_00020478*) is highlighted in the label. C, Electrophoresis of fragments amplified by RACE. 5′, 5′‐RACE; 3′, 3′‐RACE; FL, full length. D, Schematic annotation of *ROA*. E, Percentage of *ROA* distribution detected by qRT‐PCR after cell fractionation. *MALAT1* and *U6* served as positive nuclear controls, and *GAPDH* and *ACTB* served as positive cytoplasmic controls. F, Northern blot of *ROA* showing the transcript length and distribution of *ROA* in the cytoplasmic and nuclear extracts. Probe 1 targets at the sequence close to the 5′ end and probe 2 targets at the sequence close to 3′ end of *ROA*. WCL, whole cell lysate; C, cytoplasmic; N, nuclear

**FIGURE 2:**
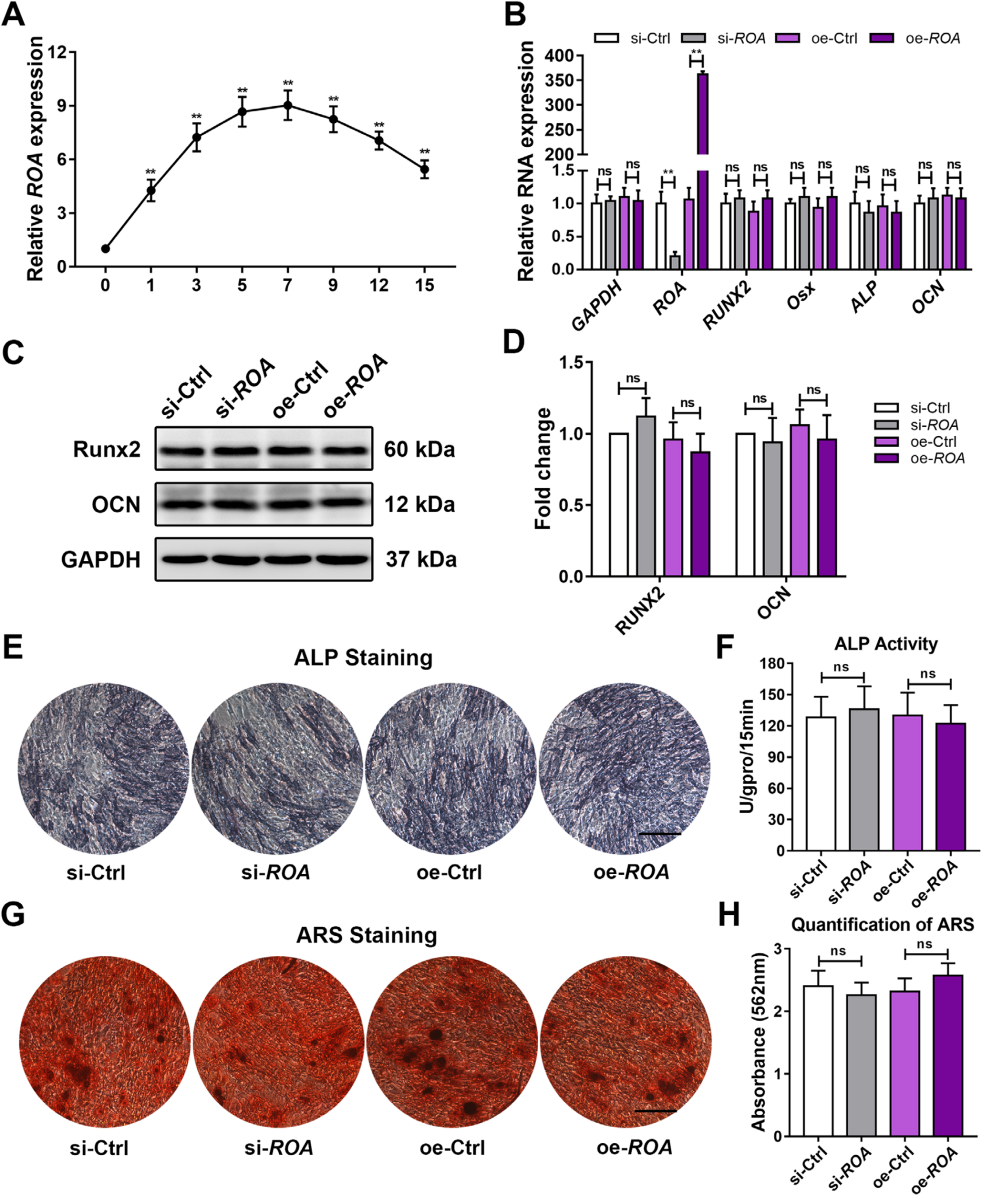
***ROA* is markedly upregulated but has little effect on MSC osteogenesis**. A, Dynamic *ROA* expression during MSC osteogenesis detected by qRT‐PCR. **, *P* < .01, compared with day 0. B, Relative expression of osteogenesis‐related genes detected by qRT‐PCR on day 3 of MSC osteogenesis after *ROA* knockdown/overexpression. C, Western blot analysis of RUNX2 and OCN on day 5. Data were normalized to GAPDH. E and F, ALP staining and activity measurement on day 7. G and H, ARS staining and quantification on day 14. Scale bar = 250 μm. The results are presented as the mean ± SD (n = 18, three independent experiments, each with six different samples). **, *P* < .01; ns, not significant, as determined by ANOVA

To investigate the roles played by lncRNAs in MSC differentiation, we performed microarray analysis on days 0 and 10 of MSCs undergoing osteogenesis. The data showed that 544 of the 20 703 analyzed lncRNAs were markedly up‐ or downregulated (fold change ≥ 2, *P* < .01; Figure 1A and B). Among these lncRNAs, we identified *TCONS_00020478* (later termed *ROA*), which was upregulated by almost nine‐fold. To confirm the transcript length and sequence of *TCONS_00020478*, we performed 5′‐ and 3′‐ rapid amplification of cDNA ends (RACE) (Figure 1C). Further sequencing and alignment with the human genome revealed it to be a 2030‐nt transcript located on human chromosome 12 with a cap and poly‐(A) tail (Figure 1D). To assess the coding ability of this transcript, we used three different prediction algorithms––Coding Potential Assessment Tool, Coding Potential Calculator 2.0, and PhyloCSF. All these algorithms labeled it as a noncoding RNA (Figure S1A‐S1C). In addition, an in vitro experiment also demonstrated that *TCONS_00020478* did not harbor protein‐coding potential (Figure S1D). Cytoplasmic/nuclear fractionation identified that *TCONS_00020478* was located in both the cytoplasm and the nucleus (Figure 1E). Northern blot analysis also showed similar results and indicated no significant difference between the nuclear and cytoplasmic forms of *TCONS_00020478* (Figure 1F). Then, we constructed both siRNAs and an overexpression Lentivirus of *TCONS_00020478* and chose the ones with the best interfering efficiency for the following experiments (Figure S2A, S2D, and S2E). To our surprise, although markedly upregulated during MSC osteogenesis (Figure 2A), knocking down or overexpressing *TCONS_00020478* had no influence on the osteogenic process of MSCs, as qRT‐PCR and western blot of the osteogenesis‐related genes, the ALP assay, and ARS staining all showed no significant differences between the knockdown/overexpression and the control groups (Figure 2B‐H).

**FIGURE 3:**
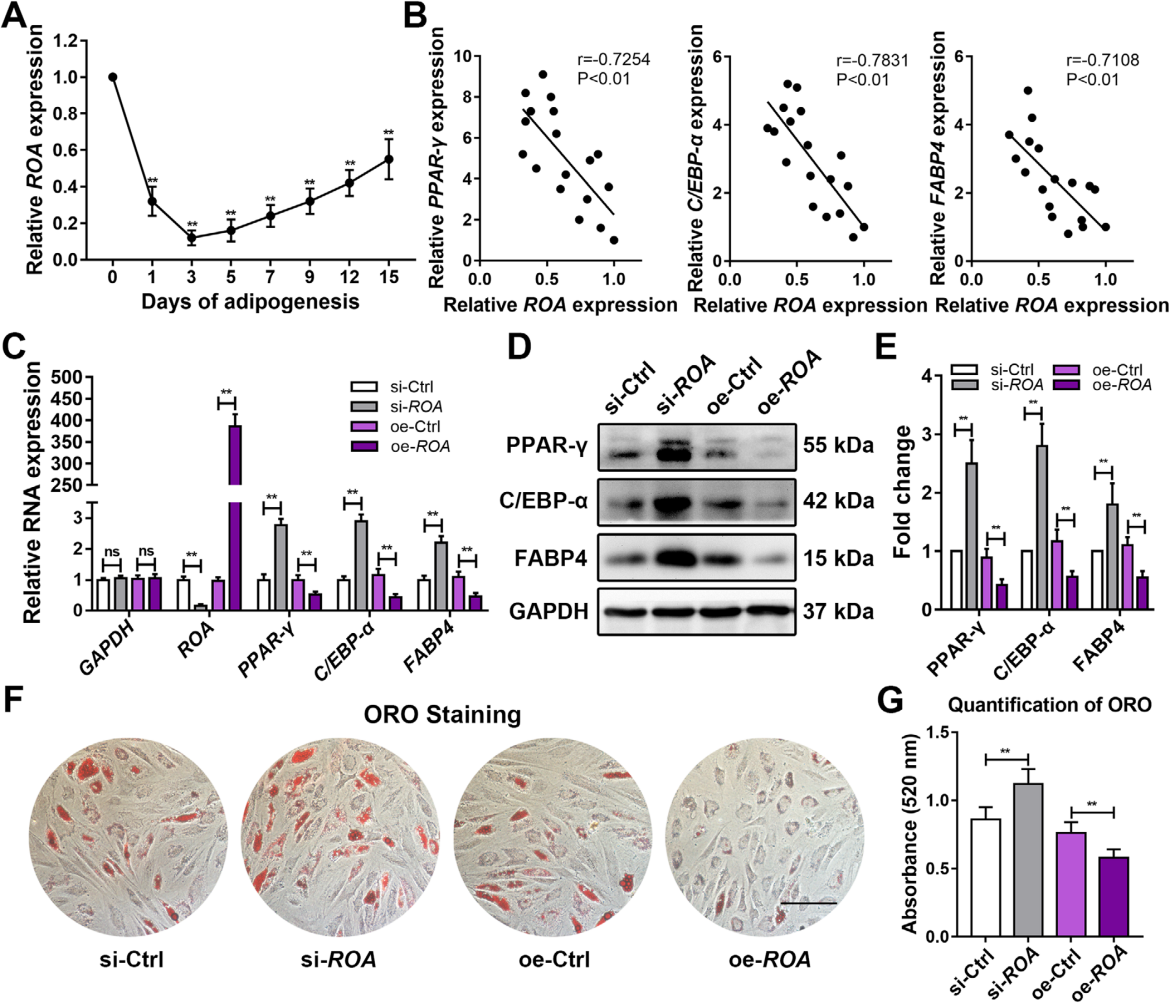
***ROA* negatively regulates MSC adipogenesis**. A, Dynamic *ROA* expression during MSC adipogenesis detected by qRT‐PCR. **, *P* < .01, compared with day 0. B, Pearson correlation analysis of *ROA* and the adipogenic markers *PPAR‐γ*, *C/EBP‐α*, and *FABP4* during MSC adipogenesis. C, Relative expression of adipogenesis‐related genes detected by qRT‐PCR on day 3 of MSC adipogenesis after *ROA* knockdown/overexpression. D and E, Western blot analysis of PPAR‐γ, C/EBP‐α, and FABP4 on day 5. Data were normalized to GAPDH. F and G, ORO staining and quantification on day 12. Scale bar = 150 μm. The results are presented as the mean ± SD (n = 18, three independent experiments, each with six different samples). **, *P* < .01; ns, not significant, as determined by ANOVA

Since osteogenesis and adipogenesis are generally recognized as two balanced fates of MSC differentiation,^5^, ^23^ we speculated that *TCONS_00020478* might play roles in the latter process to affect the former in an indirect way. With this assumption, we detected the dynamic expression of *TCONS_00020478* during MSC adipogenic differentiation and found it to be significantly downregulated, especially at the early stage (Figure 3A). Analysis of the relative expression of *TCONS_00020478* and the adipogenesis‐related genes peroxisome proliferator‐activated receptor gamma (*PPAR‐γ*), CCAAT/enhancer binding protein alpha (*C/EBP‐α*), and fatty acid binding protein 4 (*FABP4*) showed that these genes were negatively correlated during MSC adipogenesis (Figure 3B). *TCONS_00020478* knockdown in MSCs drastically increased, while *TCONS_00020478* overexpression markedly reduced the expression of *PPAR‐γ*, *C/EBP‐α*, and *FABP4*, as detected by qRT‐PCR and western blot (Figure 3C‐E). Similarly, ORO staining and quantification suggested that MSC adipogenesis was notably enhanced in the knockdown group but repressed in the overexpression group (Figure 3F and G). Together, these results indicate that *TCONS_00020478* negatively regulates the adipogenic differentiation of MSCs rather than the osteogenic differentiation. Therefore, for the convenience of annotation, we termed it repressor of adipogenesis (*ROA*). We also explored the role of *ROA* in MSC chondrogenesis but found it functionless (Figure S3).

### 3.2 *ROA* inhibits MSC adipogenesis by downregulating the expression of PTX3

**FIGURE 4:**
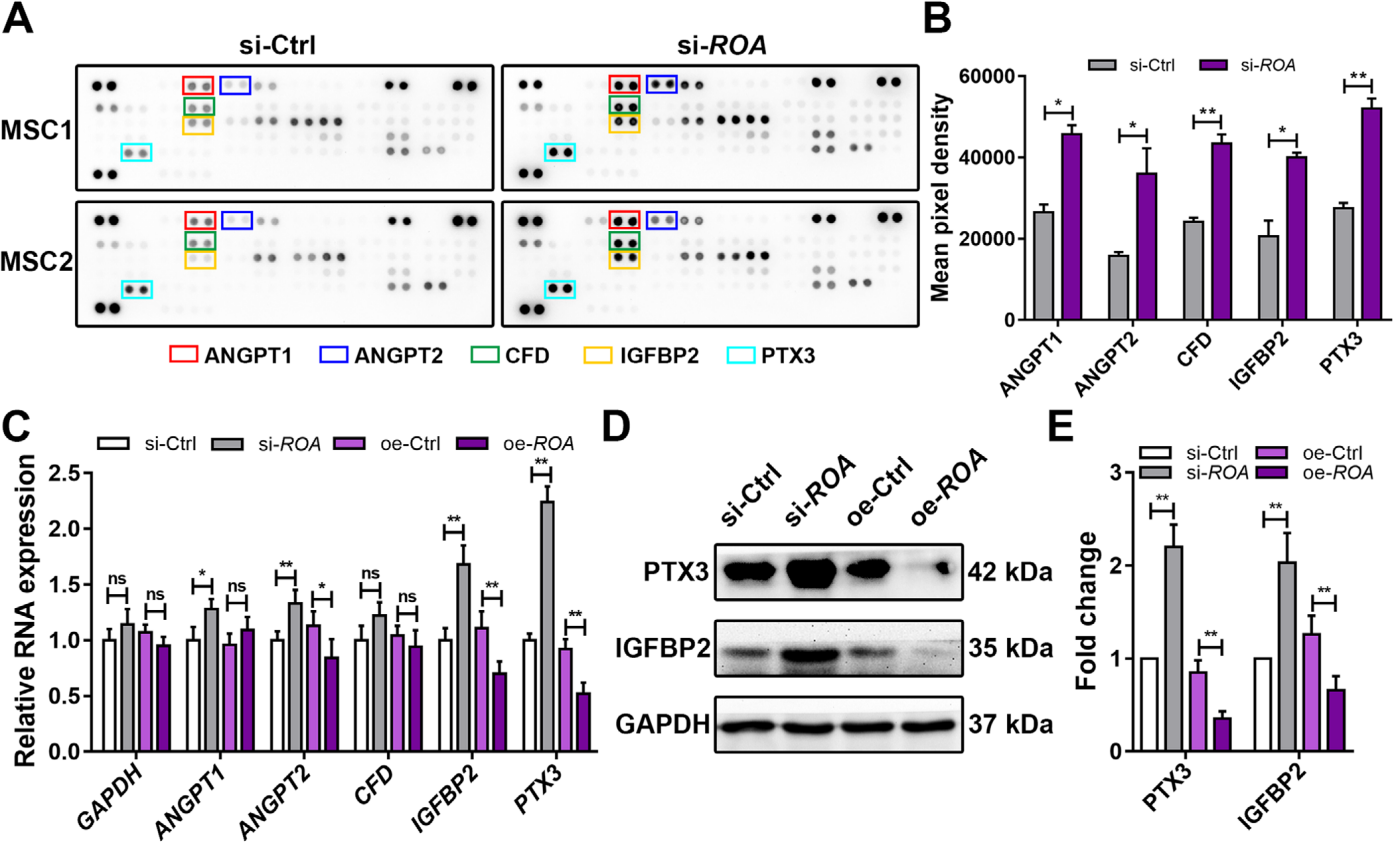
***ROA* regulates the secretion of adipokines of MSCs, especially PTX3 and IGFBP2**. A and B, Adipokines in the culture supernatant were analyzed using a Human Adipokine Array Kit on day 5 of MSC adipogenesis after *ROA* knockdown. Differentially secreted adipokines are marked with colored boxes. Data were normalized to reference spots (n = 2). C, Relative expression of *ANGPT1*, *ANGPT2*, *CFD*, *IGFBP2*, and *PTX3* determined by qRT‐PCR on day 3 of MSC adipogenesis after *ROA* knockdown/overexpression. D and E, Western blot of PTX3 and IGFBP2 on day 5. Data were normalized to GAPDH. The results are presented as the mean ± SD (n = 18, three independent experiments, each with six different samples unless stated elsewhere). *, *P* < .05; **, *P* < .01; ns, not significant, as determined by the Student's *t*‐test or ANOVA

**FIGURE 5:**
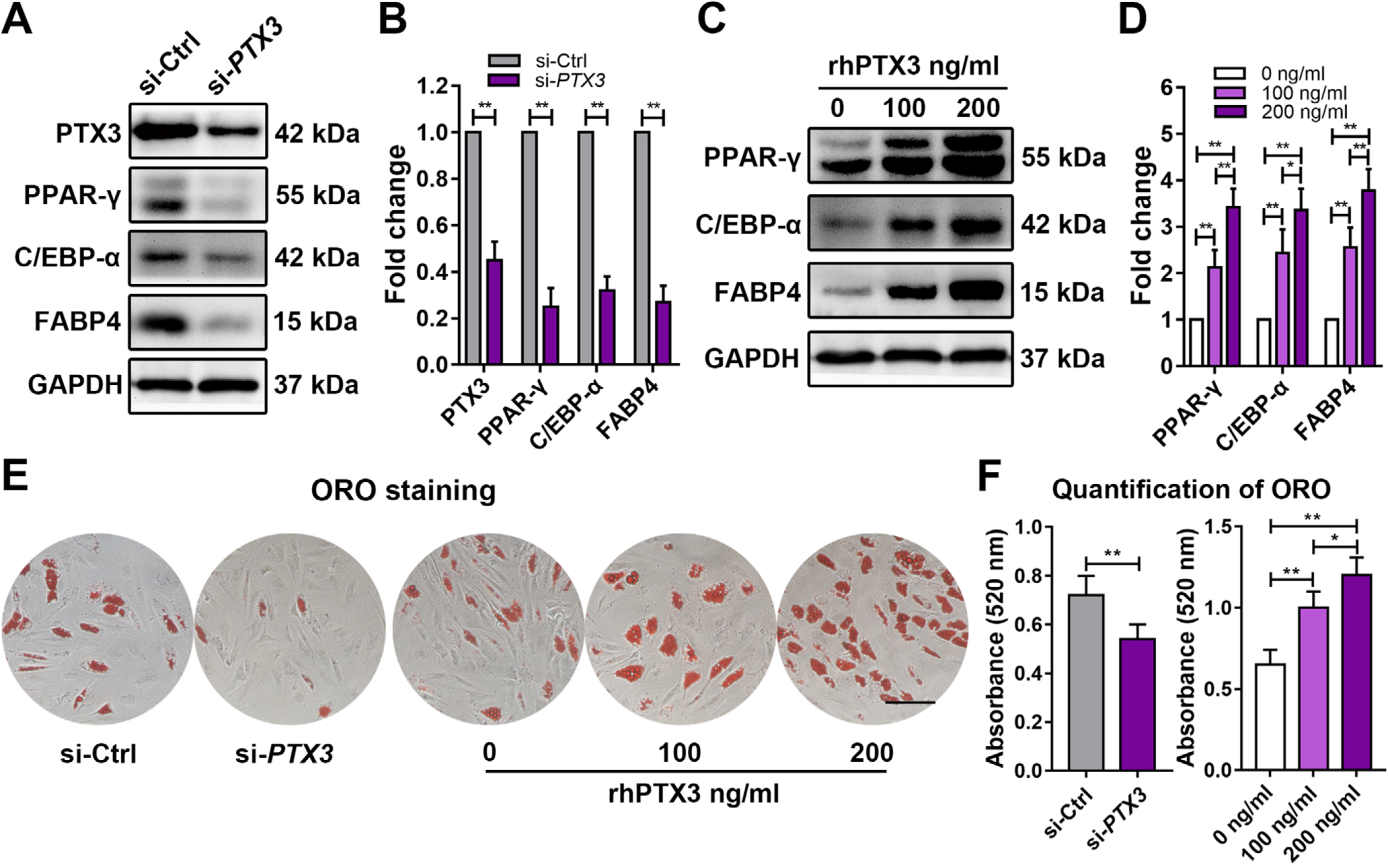
**PTX3 is an adipokine that can promote MSC adipogenesis**. A and B, Western blot of PTX3 and adipogenic markers on day 5 of MSC adipogenesis after *PTX3* knockdown. Data were normalized to GAPDH. C and D, Western blot of adipogenic markers on day 5 of MSC adipogenesis after recombinant human PTX3 (rhPTX3) stimulation. Data were normalized to GAPDH. E and F, MSC adipogenesis evaluated by ORO staining and quantification on day 12 after *PTX3* knockdown or rhPTX3 stimulation. Scale bar = 150 μm. The results are presented as the mean ± SD (n = 18, three independent experiments, each with six different samples unless stated elsewhere). *, *P* < .05; **, *P* < .01, as determined by the Student's *t*‐test or ANOVA

Accumulating evidence has shown that in response to external stimuli, MSCs can regulate their own biological behaviors, such as proliferation, differentiation, and senescence, in an autocrine/paracrine fashion.^24^, ^25^, ^26^ Based on this fact, we hypothesized that *ROA* might regulate the adipogenic process by altering the secretome of MSCs. Therefore, a total of 58 human adipokines in the culture supernatants were detected using a Human Adipokine Array Kit, and several adipokines, including angiopoietin 1 (ANGPT1), angiopoietin 2 (ANGPT2), complement factor D (CFD), insulin‐like growth factor‐binding protein 2 (IGFBP2), and pentraxin 3 (PTX3), were found to be differentially secreted after *ROA* knockdown (Figure 4A and B). The expression change of these adipokines screened by the Adipokine Array was further confirmed by qRT‐PCR and western blot using more samples (n = 18), which showed that *PTX3* and *IGFBP2* had the most significant expression changes after *ROA* knockdown/overexpression (Figure 4C‐E). We then examined the functions of these two molecules in MSC adipogenesis by either siRNA interference or exogenous PTX3/IGFBP2 stimulation. Knocking down *PTX3* led to decreased expression of the adipogenic markers *PPAR‐γ*, *C/EBP‐α*, and *FABP4* and less fat droplet formation according to western blot and ORO staining, while recombinant human PTX3 stimulation had the opposite effects in a dose‐dependent manner (Figure 5A‐F, and S2B). However, *IGFBP2* knockdown promoted MSC adipogenesis, while recombinant human IGFBP2 stimulation inhibited MSC adipogenesis, which was contrary to our expectations (Figures S2C, S4A‐S4F). Since the expression change in *PTX3* was the most prominent and its variation trend was consistent with its function, we considered PTX3 to be the crucial adipokine in *ROA*‐regulated MSC adipogenesis.

### 3.3 PTX3 stimulates MSC adipogenesis through the ERK signaling pathway

**FIGURE 6:**
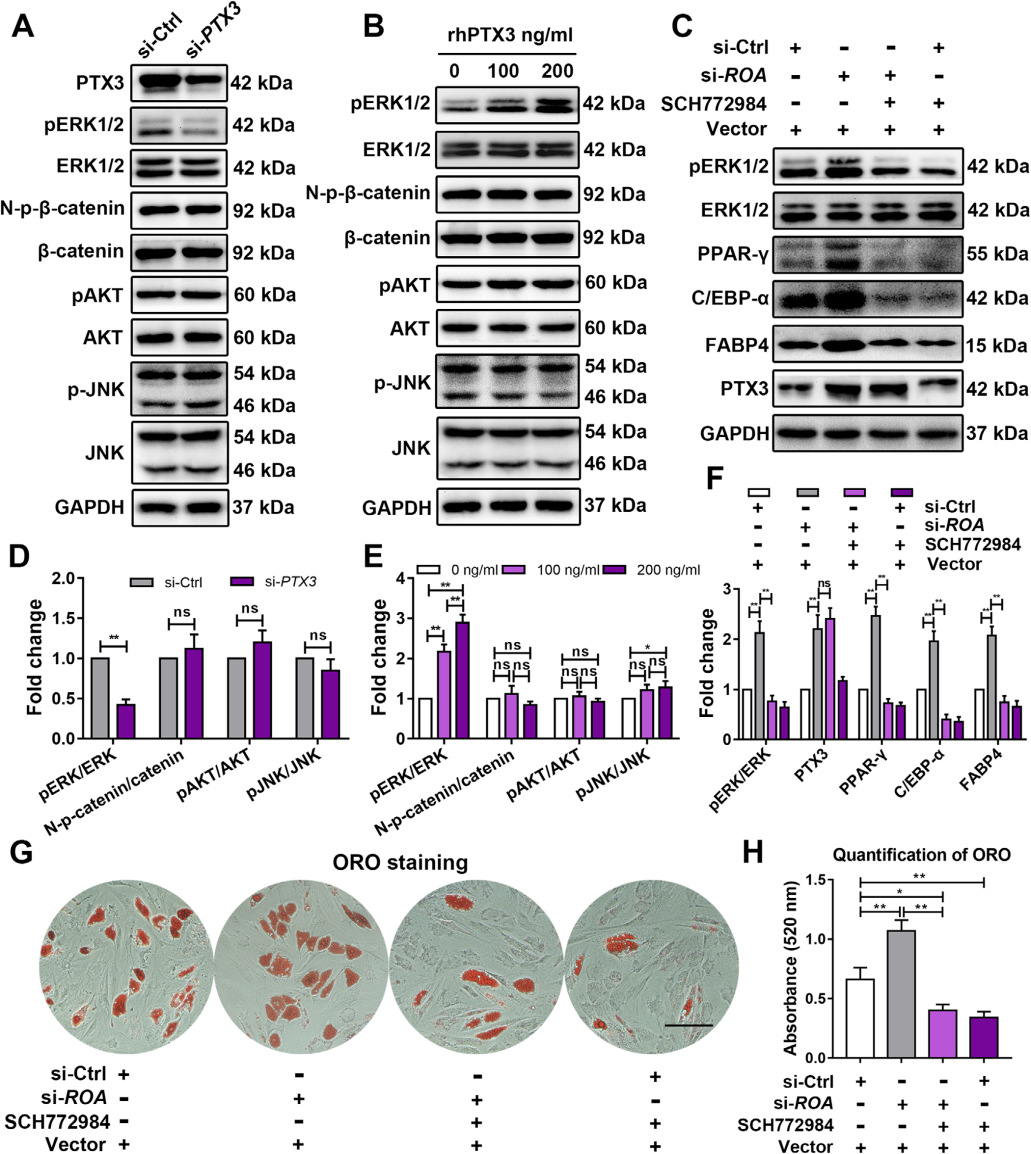
**PTX3 stimulates MSC adipogenesis through the ERK1/2 signaling pathway**. A, B, D, and E, Western blot analysis of the ERK1/2, Wnt‐β‐catenin, JNK, and AKT pathways on day 5 of MSC adipogenesis after *PTX3* knockdown or recombinant human PTX3 stimulation. Data were normalized to GAPDH. C and F, Western blot analysis of the ERK1/2 pathway and expression of PTX3 and adipogenesis‐related markers on day 5 of MSC adipogenesis after *ROA* interference and/or SCH772984 treatment. G and H, ORO staining and quantification on day 12 after *ROA* interference and/or SCH772984 treatment. Scale bar = 150 μm. The results are presented as the mean ± SD (n = 18, three independent experiments, each with six different samples). *, *P* < .05; **, *P* < .01; ns, not significant, as determined by the Student's *t*‐test or ANOVA

To investigate how PTX3 modulates the adipogenic differentiation of MSCs, we detected the activation of the Wnt/β‐catenin, JNK, PI3K‐AKT, and ERK pathways, which are the pathways reportedly involving PTX3.^27^, ^28^, ^29^, ^30^ The results showed that *PTX3* interference decreased, while rhPTX3 stimulation markedly increased the phosphorylation of ERK1/2, with no significant changes in the other pathways (Figure 6A, B, D, and E). To confirm the role played by ERK1/2 in this process and to determine the upstream and downstream relationship between ERK1/2 and PTX3, we added SCH772984, a highly selective ERK1/2 inhibitor, to MSCs after *ROA* knockdown and found that although the level of PTX3 did not show significant change, the expression of adipogenic markers was apparently downregulated (Figure 6C and F), indicating that ERK1/2 was also involved in the mechanism of *ROA*‐regulated adipogenesis and was downstream of PTX3. ORO staining and quantification also confirmed the downstream effect of the ERK1/2 pathway in this process (Figure 6G and H). Together, these data suggest that *ROA* regulates PTX3 and affects MSC adipogenesis through the ERK1/2 pathway.

### 3.4 *ROA* inhibits the transcription of *PTX3* by preventing hnRNP A1 from binding to the *PTX3* promoter

**FIGURE 7:**
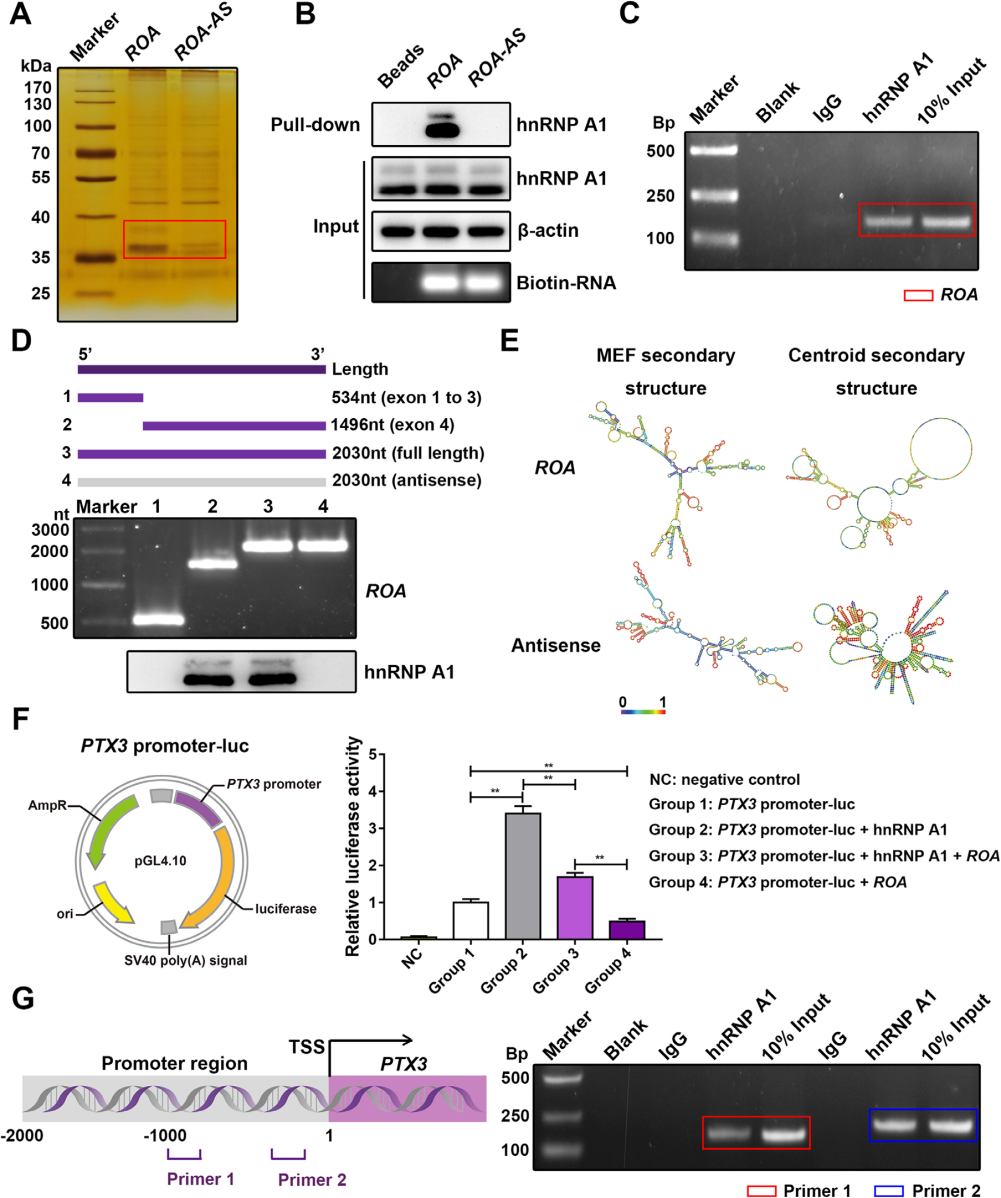
***ROA* inhibits the transcription of *PTX3* by preventing hnRNP A1 from binding to the *PTX3* promoter**. A, Silver staining of pull‐down proteins separated by SDS‐PAGE. Different bands are marked with a red box. *ROA‐AS*, antisense sequence of *ROA*. B. Western blot detection of hnRNP A1 in pull‐down proteins. Input, whole cell lysates used for incubation with biotin‐labeled RNAs; pull‐down, eluted pull‐down proteins; beads, empty magnetic bead control. C, RIP assay detection of *ROA* after hnRNP A1 immunoprecipitation. Amplified PCR products of *ROA* are marked with red box. D, Truncated fragments of *ROA* (top panel) were in vitro transcribed (middle panel) to detect the binding region of *ROA* to hnRNP A1 by RNA pull‐down. Associated hnRNP A1 was detected by western blot analysis (bottom panel). E, Secondary structure prediction of exon 4 of *ROA* and its antisense sequence. The prediction was performed using the RNAfold Webserver (http://rna.tbi.univie.ac.at/). The algorithm was based on the minimum free energy and partition function. The color scale shows the confidence of the prediction. F, The promoter region of *PTX3* was constructed into the pGL4.10 plasmid vector (left panel) for the luciferase reporter assay to detect the interaction between hnRNP A1 and the *PTX3* promoter (right panel). The results are presented as the mean ± SD (n = 9, three independent experiments, each with three different samples). **, *P* < .01, as determined by ANOVA. G, ChIP assay to detect the binding of hnRNP A1 to the *PTX3* promoter (right panel) with two designed primers (left panel). Amplified products of the *PTX3* promoter are marked with colored boxes

Next, we focused on the mechanism of *ROA*‐induced regulation of PTX3. First, we speculated that there might be a direct interaction between *ROA* and PTX3. However, the results of the RNA pull‐down assay negated our conjecture (Figure S5A). To further explore the underlying mechanism, we sent the harvested pull‐down proteins for mass spectrometry and found that hnRNP A1 was specifically enriched by *ROA* pull‐down compared with the antisense sequence of *ROA* (Figure 7A, 7B, and S5B). The interaction between *ROA* and hnRNP A1 was further verified by an RIP assay, as *ROA* was successfully enriched by hnRNP A1 immunoprecipitation (Figure 7C and S5C). To find out which region of *ROA* was responsible for the binding to hnRNP A1, we constructed the sequence near the 5′ end (exon 1 to exon 3) and 3′ end (exon 4) of *ROA* into pcDNA3.1(+) plasmid vectors, in vitro transcribed them, and examined their interaction with hnRNP A1 by the RNA pull‐down assay. The results suggested that exon 4 of *ROA* was the site for binding (Figure 7D). We also predicted the secondary structure of exon 4 and found that it had multiple stem‐loop regions and was much less crowded in structure than the antisense sequence, which may account for its protein‐binding ability (Figure 7E). Since both the *PTX3* mRNA and protein showed expression changes, we speculated that there might be a regulatory mechanism at the pretranslation level. It is reported that hnRNP A1 could promote transcription by interacting with the promoter of a gene^31^, ^32^; thus, we wondered whether hnRNP A1 could also facilitate the transcription of *PTX3* by interacting with its promoter. Therefore, we constructed the promoter area of *PTX3* (2 kb upstream of the TTS) into the pGL4.10 plasmid vector (Figure 7F). A dual‐luciferase reporter assay showed that overexpressing hnRNP A1 resulted in enhanced luciferase activity, suggesting an interaction between hnRNP A1 and the *PTX3* promoter. When *ROA* was overexpressed simultaneously, the enhanced luciferase activity was partially compromised, indicating that *ROA* could inhibit the transcription of *PTX3* by suppressing the binding of hnRNP A1 to the promoter of *PTX3* (Figure 7G). To validate the binding of hnRNP A1 to the *PTX3* promoter, we performed the ChIP assay with antibody against hnRNP A1. Compared with the IgG control, the promoter region of *PTX3*, especially the region close to the transcription starting site, was significantly enriched (Figure 7G and S5D). This region contains a large number of continuous G‐C base pairs, which is likely to form the G‐quadruplex structure with which hnRNP A1 is often reported to interact.^31^, ^32^

### 3.5 *ROA* could regulate the adipogenesis of MSC implants in vivo

**FIGURE 8:**
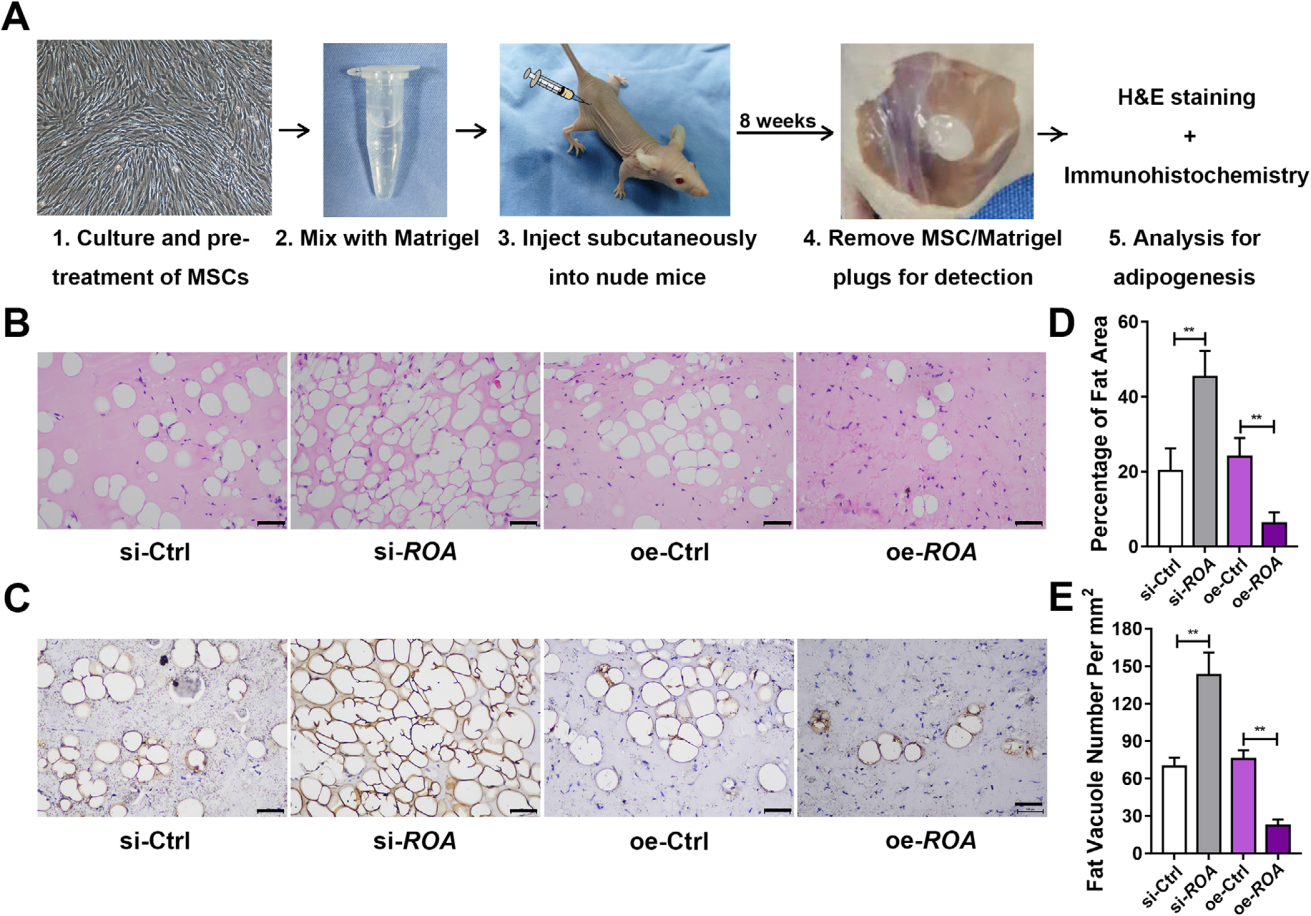
***ROA* could regulate the adipogenesis of MSC implants in vivo**. A, Schematic diagram of the in vivo MSC adipogenesis experiment. MSCs were pretreated by *ROA* knockdown or overexpression and 5 days of adipogenic induction, followed by mixing with Matrigel and subcutaneously implantation into nude mice. Adipogenesis was detected after 8 weeks. B, H&E staining showing more fat vacuoles in the knockdown group and less in the overexpression group. Scale bar = 100 μm. C, Immunohistochemistry of Perilipin‐1 showing enhanced adipogenesis in the knockdown group and inhibited adipogenesis in the overexpression group. Scale bar = 100 μm. D, Quantification of fat area percentage. E, Quantification of fat vacuole numbers

To assess the regulatory effect of *ROA* on the in vivo adipogenic differentiation of MSCs and explore its potential to improve the efficiency of tissue engineering, cells were cultured, pretreated, and mixed with Matrigel. Subcutaneous injection of the gel‐like mixture was performed on the backs of nude mice. After 8 weeks, these MSC/Matrigel plugs were removed for the assessment of adipogenesis (Figure 8A). H&E staining showed that compared with the controls, *ROA* knockdown led to more fat vacuole formation, while *ROA* overexpression significantly reduced the number and size of fat vacuoles (Figure 8B, C, and E). Immunohistochemistry staining of perilipin‐1 showed positive results in all groups, marking the success of the in vivo adipogenesis. Furthermore, the most intensive perilipin‐1 was detected in the knockdown group, and the weakest was detected in the overexpression group (Figure 8D). These results indicate that consistent with the in vitro experiment, *ROA* can effectively regulate the adipogenic differentiation of MSCs on Matrigel scaffolds in vivo.

## 4 DISCUSSION

**FIGURE 9:**
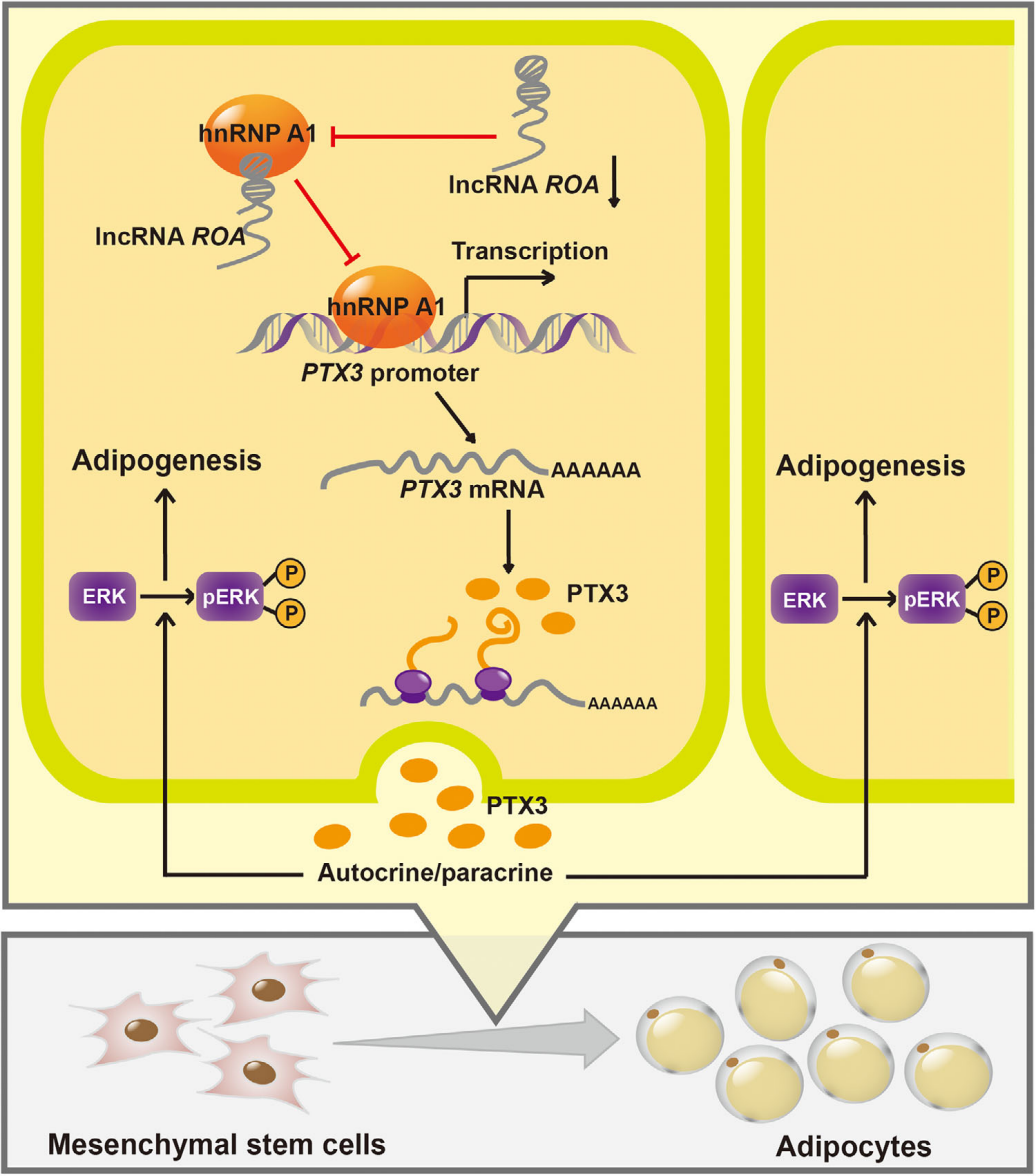
**Graphical representation of *ROA*‐regulated MSC adipogenesis**. Downregulation of *ROA* during MSC adipogenesis decreases the interaction between *ROA* and hnRNP A1 and increases the binding of hnRNP A1 to the *PTX3* promoter, resulting in increased expression of the key autocrine/paracrine factor PTX3 and activation of the downstream ERK1/2 pathway, which finally promotes MSC adipogenesis

In the present study, we identified a lncRNA termed repressor of adipogenesis (*ROA*) that is markedly upregulated in MSC osteogenesis and downregulated in MSC adipogenesis. We found that although the level of *ROA* changes in both processes, it only functions to inhibit MSC adipogenesis without affecting the osteogenic process. Mechanistically, we revealed that *ROA* inhibited MSC adipogenesis by downregulating the expression of the key autocrine/paracrine factor PTX3 and the downstream ERK pathway. This regulation is achieved through transcription inhibition by detaining hnRNP A1 to prevent its interaction with the *PTX3* promoter (Figure 9). Animal experiments also showed that *ROA* could regulate the adipogenic differentiation of MSCs on Matrigel scaffolds in vivo.

MSCs are pluripotent stem cells present in multiple tissues and are the precursors of osteoblasts, chondroblasts, and adipocytes.^1^ Osteogenesis and adipogenesis are two major directions of MSC lineage commitment and are generally thought to have an inverse relationship and restrict each other.^5^, ^23^ The process of lineage commitment is under fine regulation of multiple intrinsic and extrinsic factors, including lncRNAs. Previous research has reported that lncRNA *HoxA‐AS3* could act as a dual regulator in MSC differentiation; an elevation of its level enhances adipogenesis, while a decrease in its expression promotes osteogenesis.^33^ In our study, we found that *ROA* was upregulated in MSC osteogenesis but downregulated in MSC adipogenesis, which indicated that *ROA* might be another molecule participating in the balance between MSC osteogenesis and adipogenesis. However, further experiments revealed that *ROA* inhibited only MSC adipogenesis without affecting the osteogenic process, leaving endless questions about its role in MSC osteogenesis.

Interestingly, we previously found another lncRNA termed *lncRNA‐OG* that could regulate MSC osteogenesis by interacting with hnRNP K and promoting the expression of downstream BMP family proteins.^34^ We performed sequence alignment of these two lncRNAs and the human genome and discovered that they are located at the same gene locus and are two different transcripts of the same gene. These two transcripts share a 448 nt common sequence near the 5′ end but are completely different in terms of the sequence of the 3′ end, which may account for their different functions. In addition, our researches did demonstrate that the functional regions of both *lncRNA‐OG* and *ROA* were located in exon 4, the region where their sequences differ, further supporting this hypothesis. It has been reported that alternative splicing is an economic method of using limited gene loci to give rise to different transcripts with similar or opposite functions.^35^, ^36^, ^37^ For example, the alternative splicing of *LINC00477* can produce two isoforms with different tumor suppression abilities.^38^ The two functionally different transcripts in our studies indicate that alternative splicing at this gene locus may be vital in determining the fate of MSCs. Different microenvironments induce different forms of splicing, which eventually leads MSCs toward different differentiation outcomes. Further exploration is needed to elucidate how this alternative splicing is organized.

Autocrine/paracrine is an effective way to establish communication between adjacent cells and harmonize the collective behavior of cell populations. It has been reported that in response to external stimuli, MSCs can regulate their own biological behaviors, including adipogenesis, in an autocrine/paracrine manner.^39^, ^40^ Our study revealed that *ROA* also takes autocrine/paracrine as the downstream mechanism and serves as a potent modulator of the secretome of MSCs. After *ROA* interference, the secretion profile of 58 adipokines changed greatly, especially the levels of PTX3 and IGFBP2. PTX3 is a component of humoral innate immunity.^41^ It has been reported that PTX3 can promote the adipogenesis of 3T3‐L1 cells,^42^ which drew our attention. Therefore, we examined the function of PTX3 in MSC adipogenesis and found it to be promotive because *PTX3* interference/recombinant human PTX3 stimulation correspondingly resulted in impaired/enhanced MSC adipogenesis. Since the expression change in *PTX3* was the most prominent and its variation trend was consistent with its function, we recognized PTX3 as the crucial secreting factor in *ROA*‐regulated MSC adipogenesis. IGFBP2 is generally thought to inhibit adipogenesis by binding to and attenuating the effect of IGFs.^43^, ^44^ Our experiments also suggested that IGFBP2 functions as an inhibitor in MSC adipogenesis, despite showing an apparent upregulation after *ROA* knockdown. We suspect that this contradiction may be explained by negative feedback. Owing to its versatile nature, MSCs have been widely employed in regenerative and translational medicine in the last few years, and there is accumulating evidence suggesting that MSCs exert their therapeutic effects by releasing secreting factors, either as free soluble proteins or microstructured extracellular vesicles.^45^, ^46^, ^47^ Since *ROA* can effectively modulate the secretion spectrum of MSCs, it may serve as a critical target for modifying MSCs in clinical use.

Controversies over the role of MAPK in adipogenesis have existed for a long time.^48^ Our experiments showed that PTX3 could promote MSC adipogenesis by activating the ERK pathway. Several studies also reported similar results indicating that ERK activation could facilitate MSC adipogenesis.^49^, ^50^ However, more studies have shown that on the scale of MSC osteogenesis and adipogenesis, activation of the ERK pathway stands at the osteogenic side, while final adipogenic differentiation requires dephosphorylation of ERK.^51^, ^52^, ^53^ Concerning the exact role played by ERK in MSC adipogenesis, we suppose that the timepoint could explain this question. It has been reported that the adipogenesis of MSCs can be roughly divided into two phases. The first phase is the lineage commitment phase, during which pluripotent MSCs differentiate into preadipocytes and undergo several rounds of cell expansion. The second phase is the differentiation phase, during which preadipocytes become spherical mature adipocytes.^54^, ^55^, ^56^ Activation of ERK is required during the initiation stage, at which lineage commitment and proliferation of preadipocytes occur, but ERK needs to be dephosphorylated when differentiation proceeds.^57^, ^58^ Since the downregulation of *ROA* was more prominent at the beginning of the adipogenic process, the timepoint we selected for pathway detection was quite early and might still be at the first phase of MSC adipogenesis. In addition, there is a study showing that in the adipogenic differentiation of MSCs, high levels of PTX3 can be expressed at the very early stage,^59^ which is consistent with our results and indicates that detecting ERK at an early timepoint may explain the mechanism through which PTX3 exerts its effect better than at a late timepoint. As adipogenesis is a sequentially ordered process involving multiple signaling cascades, we believe that the regulatory value of *ROA* on PTX3 and ERK is mostly exhibited at the initial stage of MSC adipogenesis.

Heterogeneous nuclear ribonucleoproteins are a large family of RNA‐binding proteins that participate in multiple aspects of RNA metabolism, including alternative splicing, mRNA stabilization, and transcriptional regulation.^60^ Among hnRNPs, hnRNP A1 is the most ubiquitously expressed and best studied, and it has been reported to participate in diverse processes in the development and differentiation of living organisms.^61^ The function of hnRNP A1 in transcriptional regulation has gradually been revealed in recent years. For example, Cogoi et al found that hnRNP A1 could bind to the promoter area of the human *KRAS* gene and lead to its activation in pancreatic cancer cells.^31^ Nishikawa et al reported that hnRNP A1 could interact with the *TRA2B* promoter and stimulate its transcription in human colon cancer cells.^32^ In this study, we discovered that hnRNP A1 could also bind to the *PTX3* promoter and promote its transcription. The interaction between *ROA* and hnRNP A1 prevented its binding to the *PTX3* promoter, compromising its transcriptional regulatory activity and ultimately resulting in decreased *PTX3* transcription and impaired MSC adipogenesis. These results add new insights to the transcriptional regulator mechanism of hnRNP A1 and further substantiate the powerful and diversified capacities, as well as the unshakable position of the hnRNP family in controlling cell events.

Tissue engineering is a highly interdisciplinary research field driven by the goal of restoring, replacing, and regenerating defective tissues.^62^, ^63^ Owing to their easy availability, low immunogenicity, and great potential for differentiation, MSCs have become a promising cell choice in tissue engineering.^64^, ^65^, ^66^ To date, adipogenic differentiation has been applied for breast augmentation and restoration of soft tissue defects in esthetic and plastic areas.^9^, ^10^, ^11^ In our in vivo adipogenesis experiment, we observed more fat vacuole formation and perilipin‐1 expression in the *ROA* knockdown group than in the control group, indicating an improved grafting efficiency. Our animal experiment was a new attempt to apply MSC adipogenesis in tissue engineering, in which we demonstrated that pretreatment with *ROA* effectively controlled the efficiency of MSC adipogenesis in vivo.

Overall, our findings support a model in which the lncRNA *ROA* acts as a potent regulator of MSC adipogenesis through the *ROA*‐hnRNP A1‐PTX3‐ERK axis and provide further evidence of a potential target for modifying MSCs in tissue engineering and other application fields. However, our study still has some limitations. Important unanswered questions remain. For example, what is the upstream regulatory mechanism of *ROA*? How is the alternative splicing of *ROA* achieved? And which target on the *ROA*‐hnRNP A1‐PTX3‐ERK axis is optimal for regulation in regard to application value? All these questions need to be explored through more in‐depth studies.

## 5 CONCLUSIONS

In this study, we identified a new lncRNA transcript termed repressor of adipogenesis (*ROA*) that can effectively inhibit the adipogenesis of MSCs. Mechanistically, ROA inhibits MSC adipogenesis by impeding hnRNP A1 from binding to the promoter of *PTX3*, thus downregulating the expression of the key autocrine/paracrine factor PTX3 and the downstream ERK pathway. Our in vivo adipogenesis model also suggested that by modulating *ROA*, the grafting efficiency can be effectively controlled. *ROA* may serve as a critical target for modifying MSCs in tissue engineering.

## FUNDING INFORMATION

This work was supported by the National Natural Science Foundation of China [81971518], the Fundamental Research Fundus for the Central Universities of China [19ykpy01], the Shenzhen Key Medical Discipline Construction Fund [ZDSYS20190902092851024], and the Health Welfare Fund Project of Futian District [FTWS2019020].

## CONFLICT OF INTEREST

The authors declare no conflict of interest.

## ETHICS APPROVAL AND CONSENT TO PARTICIPATE

This study was approved by the ethics committee of The Eighth Affiliated Hospital, Sun Yat‐sen University (Shenzhen, China). Written informed consent was obtained from all participants. The animal experiment was approved by the Animal Ethical and Welfare Committee of The Eighth Affiliated Hospital, Sun Yat‐sen University.

## AUTHOR CONTRIBUTIONS

Yiqian Pan and Zhongyu Xie designed the study. Yiqian Pan, Shuizhong Cen, and Ming Li performed the experiments. Shuizhong Cen, Su'an Tang, and Wenhui Yu interpreted the data. Guiwen Ye, Zhaofeng Li, and Jinteng Li prepared the figures and tables. Yiqian Pan, Ming Li, and Wenjie Liu wrote the manuscript. Zhongyu Xie, Guan Zheng, and Jinteng Li edited the manuscript. Peng Wang, Yanfeng Wu, and Huiyong Shen supervised the study. All authors read and approved the final manuscript.

## CONSENT FOR PUBLICATION

The authors agree to the publication of all the data involved in this article. No data from other entities are used in this study.

## Supporting information

Supporting Information

Supporting Information

Supporting Information

## Data Availability

The analyzed data sets generated during the study are available from the corresponding author on reasonable request.
