# Supplementary material for: Long noncoding RNA repressor of adipogenesis negatively regulates the adipogenic differentiation of mesenchymal stem cells through the hnRNP A1‐PTX3‐ERK axis
Source: Clin Transl Med. 2020 Nov 8;10(7):e227. doi: 10.1002/ctm2.227 (PMC7648959; doi:10.1002/ctm2.227)
Supplement: Supplementary file 1 — Supporting Information [file CTM2-10-e227-s001.pdf]

Supplementary Figures S1 to S4

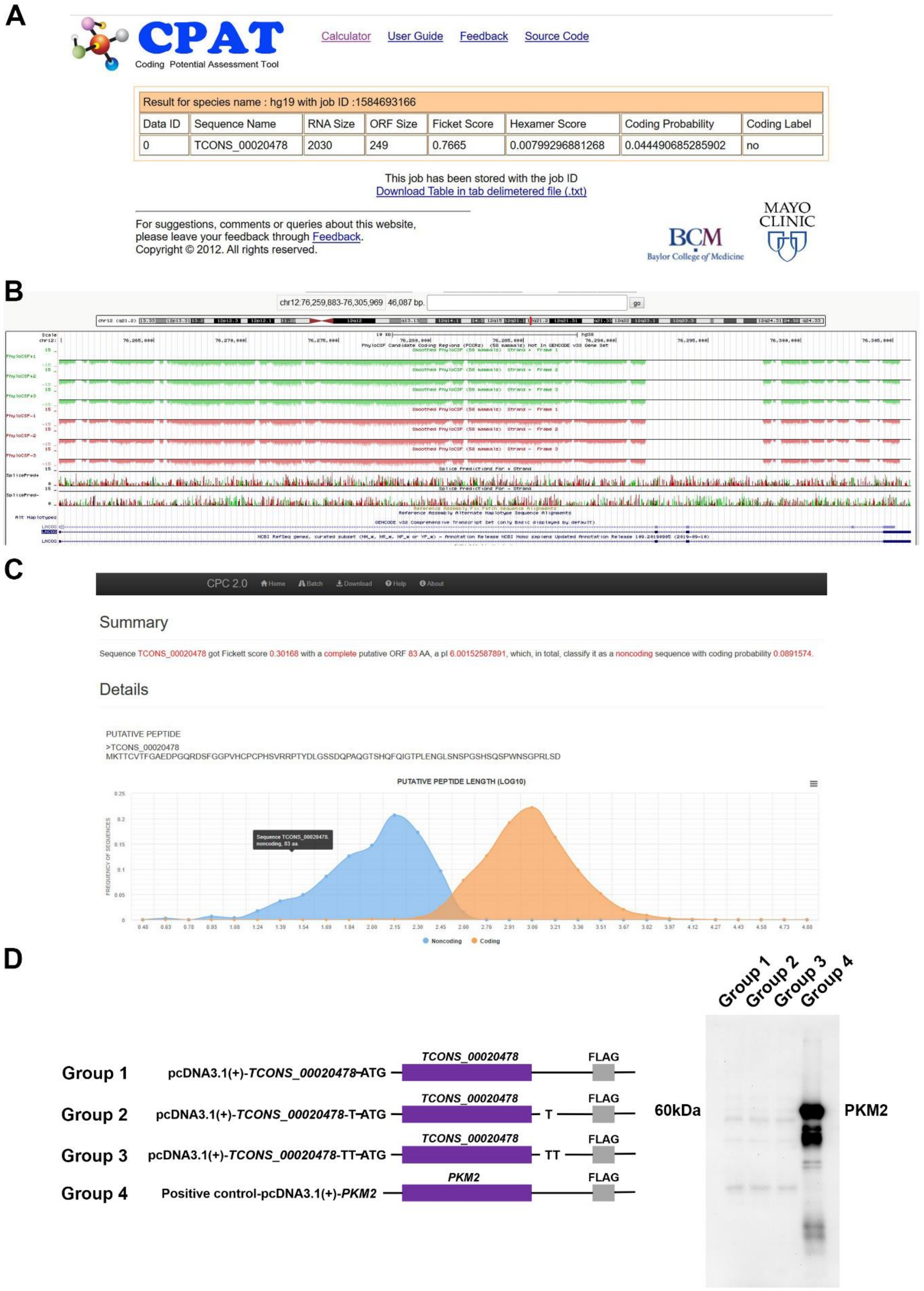

FIGURE S1

A to C, Prediction of the coding ability of *TCONS_00020478* using the online tools Coding Potential Assessment Tool, PhyloCSF, and Coding Potential calculator all showed little protein-coding possibility of *ROA*.

D, An in vitro expression assay confirmed that *TCONS_00020478* had no protein-coding ability.

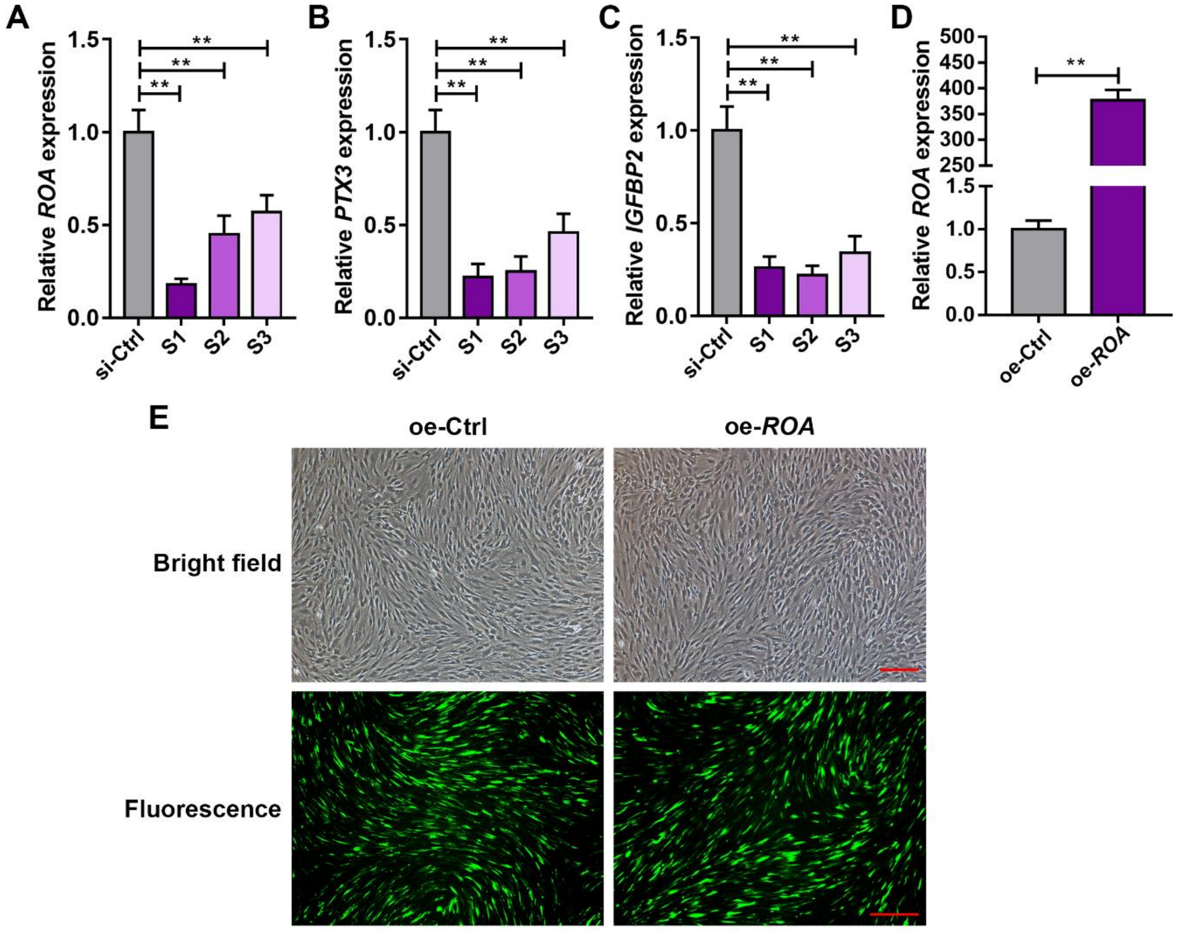

FIGURE S2

A to C, Knockdown efficiency of siRNA sequences designed for *ROA*, *PTX3* and *IGFBP2* detected by qRT-PCR on day 3 after transfection. The ones with the best interfering efficiency were chosen for the following experiments.

D, Overexpression efficiency of *ROA* detected by qRT-PCR on day 3 after Lentivirus transfection.

E, Bright field and fluorescent images showing *ROA* was evenly overexpressed among cells and did not have an apparent influence on cell viability or morphology. Scale bar = 150 μm. The results are presented as the mean ± SD (n = 18, three independent experiments, each with six different samples). **, $p<0.01$, as determined by ANOVA.

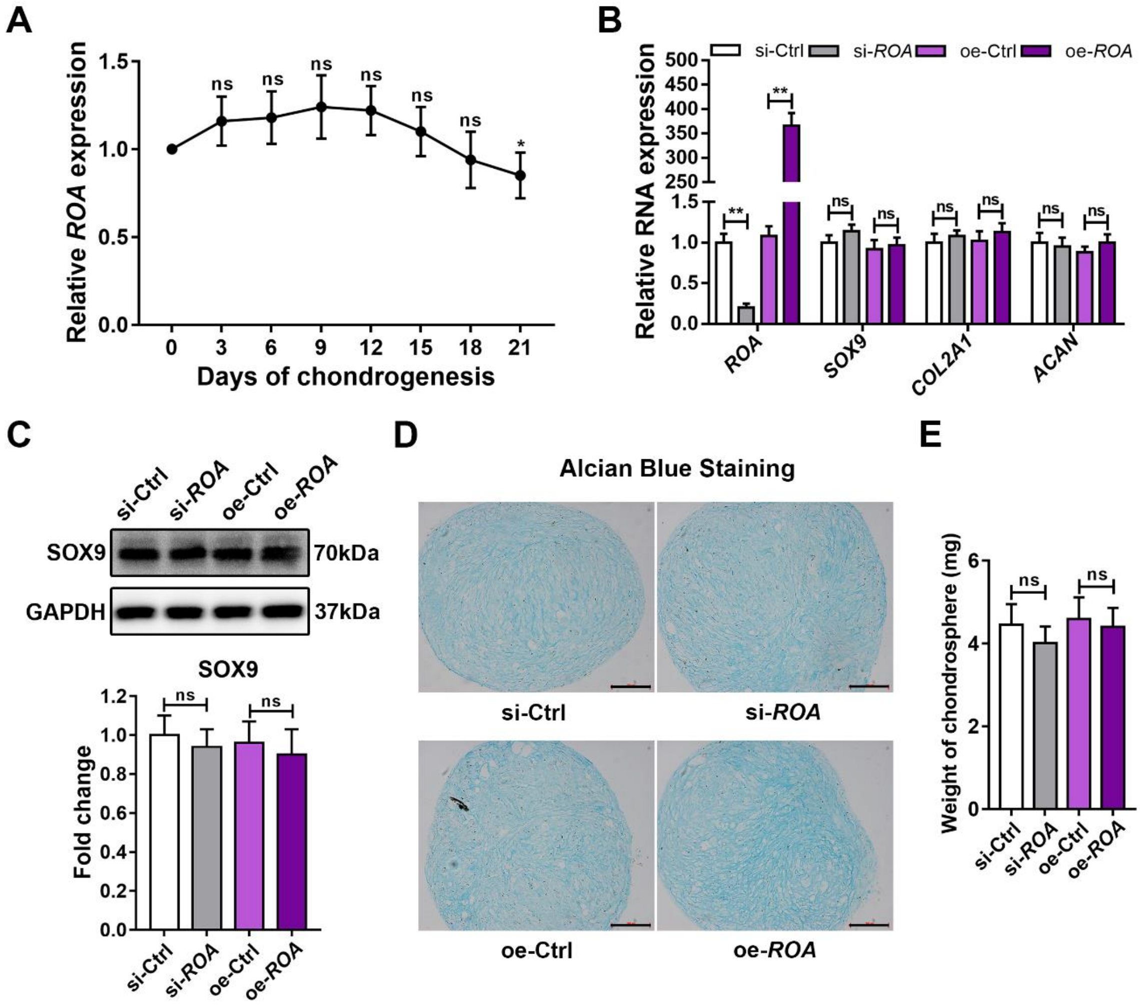

FIGURE S3

A, Dynamic *ROA* expression during MSC chondrogenesis detected by qRT-PCR. *, p<0.05, ns, not significant, compared to day 0.

B, Relative expression of chondrogenesis-related genes detected by qRT-PCR on day 3 of MSC adipogenesis after *ROA* knockdown/overexpression.

C, Western blot of SOX9 on day 7. Data were normalized to GAPDH.

D, Alcian blue staining on day 21. Scale bar = 150 μm.

E, Quantification of chondrosphere weight on day 21.

The results are presented as the mean ± SD (n = 18, three independent experiments, each with six different samples). **, p<0.01; ns, not significant, as determined by ANOVA.

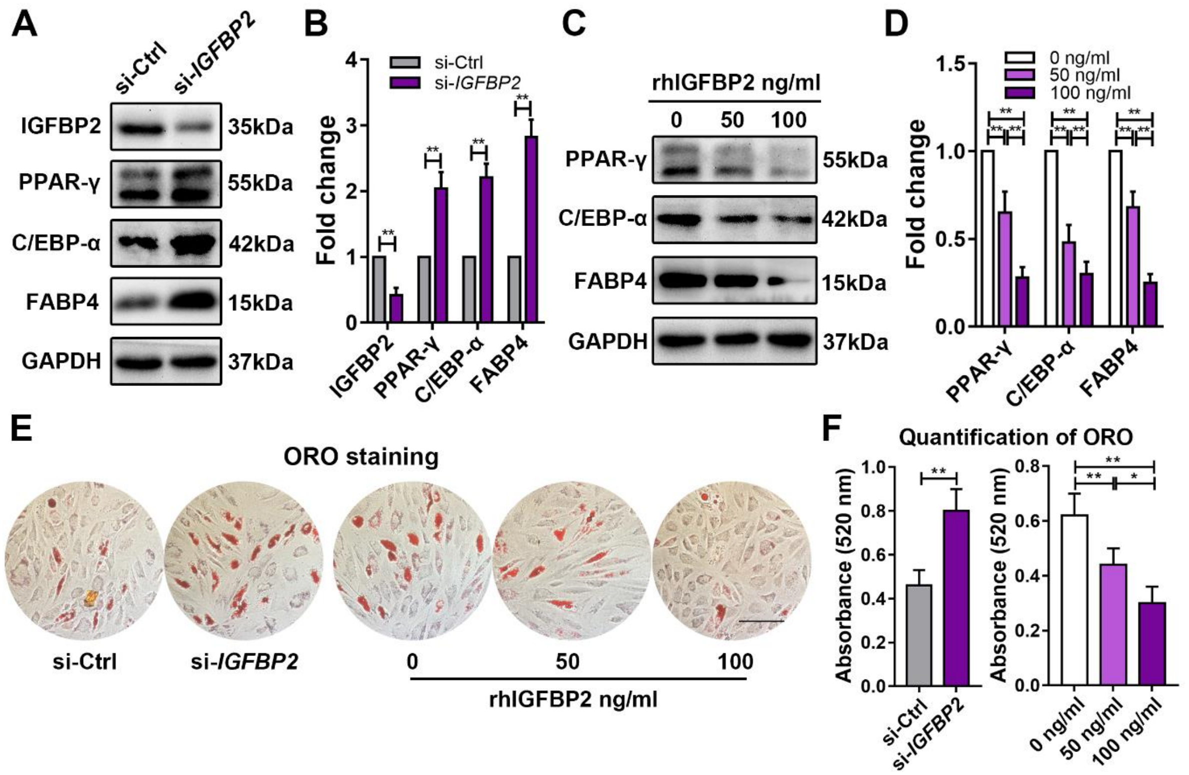

FIGURE S4

A and B, Western blot of IGFBP2 and adipogenic markers on day 5 of MSC adipogenesis after *IGFBP2* knockdown. Data were normalized to GAPDH.

C and D, Western blot of adipogenic markers on day 5 of MSC adipogenesis after recombinant human IGFBP2 (rhIGFBP2) stimulation. Data were normalized to GAPDH.

E and F, MSC adipogenesis evaluated by ORO staining and quantification on day 12 after *IGFBP2* knockdown or rhIGFBP2 stimulation. Scale bar = 150 μm.

The results are presented as the mean ± SD (n = 18, three independent experiments with six different samples). *, p<0.05; **, p<0.01, as determined by Student's t test or ANOVA.

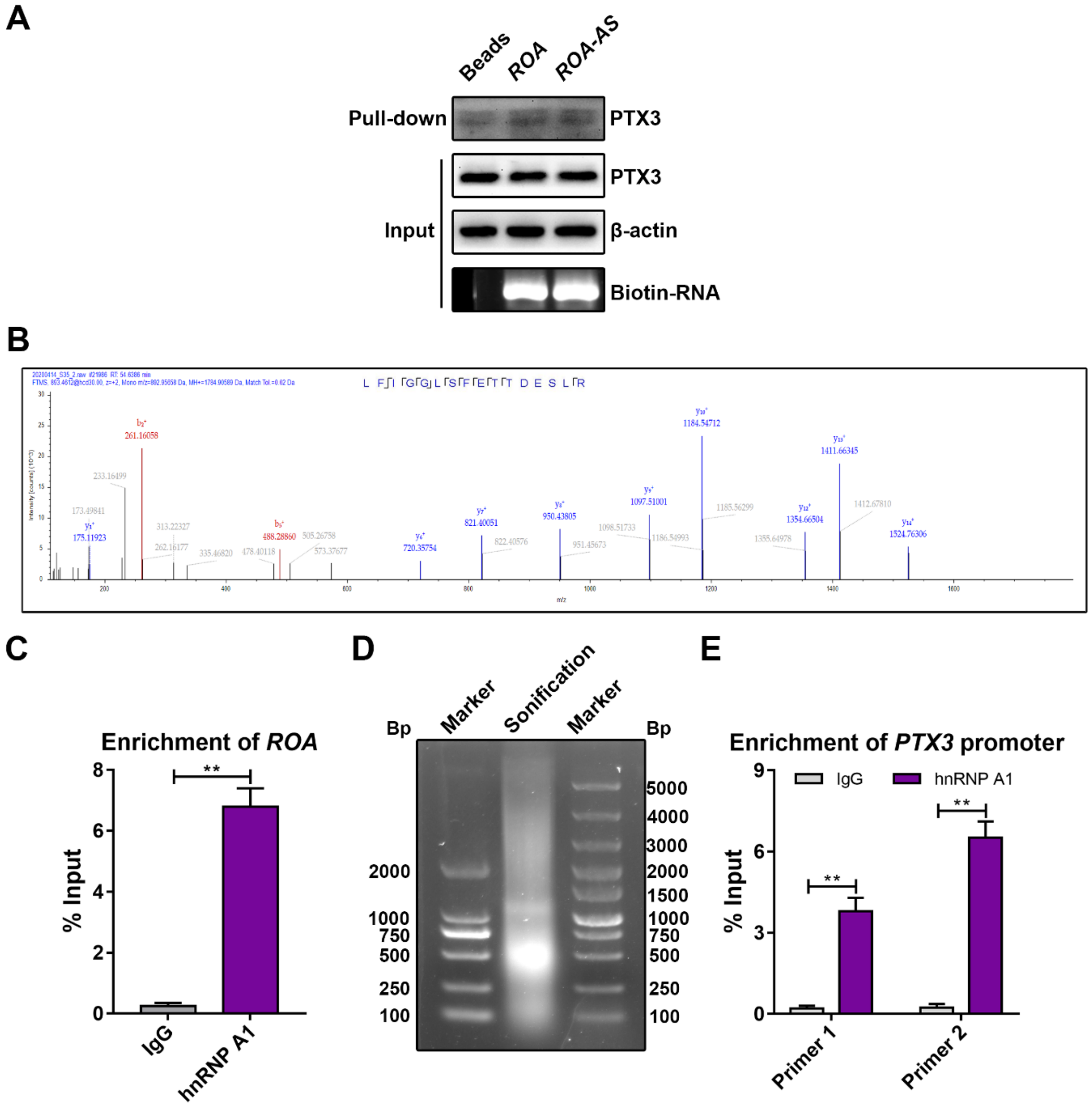

FIGURE S5

A, Western blotting showing PTX3 was not enriched by pull-down with biotinylated *ROA* compared to *ROA-AS* or empty magnetic beads control.

B, The peptide sequence of hnRNP A1 was detected in the pull-down proteins by mass spectrometry. Red peaks indicate secondary fragments close to the N-terminal and blue to the C-terminal.

C, Quantification of the RIP assay.

D, Agarose gel electrophoresis of ChIP sonification product. Most fragments were of 200~1000 bp in length.

E, Quantification of the ChIP assay.
