# Supplementary material for: Long noncoding RNA repressor of adipogenesis negatively regulates the adipogenic differentiation of mesenchymal stem cells through the hnRNP A1‐PTX3‐ERK axis
Source: Clin Transl Med. 2020 Nov 8;10(7):e227. doi: 10.1002/ctm2.227 (PMC7648959; doi:10.1002/ctm2.227)
Supplement: Supplementary file 2 — Supporting Information [file CTM2-10-e227-s002.docx]

**Supplementary Table S1. Primers used for 5’- and 3’- RACE of lncRNA *ROA*.**

| **Primer** | **Sequence (5’-3’)** |
| --- | --- |
| 5’GSP-R1 | CAGACACAGCCATGTTCCCATTCAC |
| 5’GSP-R2 | GCTCCACTTCACTGTCGGTCTCCCT |
| 3’GSP-F1 | GTATCTTCTGTGGCCCCATGACTAATTTCG |
| 3’GSP-F2 | GCCTCACAGGTTGGCTGTCTTGGCTC |
| UPM-L | CTAATACGACTCACTATAGGGCAAGCAGTGGTATCAACGCAGAGT |
| UPM-S | CTAATACGACTCACTATAGGGC |
| FL-F | GATCTGGCAGGGCTTTAAAG |
| FL-R | GACACGACGCCAGCCACGCC |

Abbreviation: 5’GSP-R, reverse gene specific primer for 5’-RACE; 3’GSP-F, forward gene specific primer for 3’-RACE; UPM-L, long universal primer mix; UPM-S, short universal primer mix; FL-F, forward primer for full length lncRNA *ROA*; FL-R, reverse primer for full length lncRNA *ROA*.

**Supplementary Table S2. Probes used for northern blot**

| **Probe** | **Sequence (5’-3’)** |
| --- | --- |
| Probe 1^*^ | TCCAGGGGCTCTGGGAGTGGCTGCCGGGTGAGTTGGACAGTCCATTTTCCAGTGGGGTCCCGATTTGAAATTGGTGAGATGTTCCTTGAGCTGGTTGGTCTGAGGACCCGAGGTCGTAGGTGGGTCTCCTCACAGAGTGAGGGCAAGGACAGTGGACTGGTCCCCCAAAGGAGTCTCTCTGACCTGGGTCTTCAGCACCAAATGTCACGCATGTCGTTTTCATCAGTTGTTTTCTTAGAGCTCTGGATATTTCTCTTGTAAGAGGAACAATCTGTGCAATTACAGTTCTGTAAAATACAG |
| Probe 2^**^ | TTGTTTCTCCACCTTCCCCTAGGGCCTTGTCCTTTGCTGCACAAAGTCGGGCTGCAGACACAGCCATGTTCCCATTCACAGATAAGACAAAGAGTGGAAATGGAGGGCGGTCATATTTTTTTTTAAACCAATGAACCCAAGTGCATACATCACTTCCACTTACATTCCACTGTCACGTGGCCACACCTGGCCACAAGGGTGACTAAGAGGTGCAGTCTTTAGGTAGGTAGCCATAGGCCCAGCTAAATGCTGTTCTTCTGGAAGAAAGGGAGAATGGCCTTTGGTGAATTCATGGCAGGC |
| β-actin | GGGCGACGTAGCACAGCTTCTCCTTAATGTCACGCACGATTTCCCGCTCGGCCGTGGTGGTGAAGCTGTAGCCGCGCTCGGTGAGGATCTTCATGAGGTAGTCAGTCAGGTCCCGGCCAGCCAGGTCCAGACGCAGGATGGCATGGGGGAGGGCATACCCCTCGTAGATGGGCACAGTGTGGGTGACCCCGTCACCGGAGTCCATCACGATGCCAGTGGTACGGCCAGAGGCGTACAGGGATAGCACAGCCTGGATAGCAACGTACATGGCTGGGGTGTTGAAGGTCTCAAACATGATCT |

*Probe 1 targets at nucleotide 101 to 400 of lncRNA *ROA*.

**Probe 2 targets at nucleotide 601 to 900 of lncRNA *ROA*.

**Supplementary Table S3. Primers used for qRT-PCR.**

| **Gene** |  | **Primer sequence (5’-3’)** | |
| --- | --- | --- | --- |
| *GAPDH* | Forward | | ACAACTTTGGTATCGTGGAAGG |
|  | Reverse | | GCCATCACGCCACAGTTTC |
| *ACTB* | Forward | | CATGTACGTTGCTATCCAGGC |
|  | Reverse | | CTCCTTAATGTCACGCACGAT |
| *U6* | Forward | | CTCGCTTCGGCAGCACA |
|  | Reverse | | AACGCTTCACGAATTTGCGT |
| *MALAT1* | Forward | | GTCATAACCAGCCTGGCAGT |
|  | Reverse | | CGAAACATTGGCACACAGCA |
| LncRNA *ROA* | Forward | | GTCCCATAGAGGGAGACCGA |
|  | Reverse | | TCATGGGGCCACAGAAGATAC |
| *ALP* | Forward | | ACTGGGGCCTGAGATACCC |
|  | Reverse | | TCGTGTTGCACTGGTTAAAGC |
| *RUNX2* | Forward | | TCAACGATCTGAGATTTGTGGG |
|  | Reverse | | GGGGAGGATTTGTGAAGACGG |
| *OSX* | Forward | | CCTCTGCGGGACTCAACAAC |
|  | Reverse | | AGCCCATTAGTGCTTGTAAAGG |
| *OCN* | Forward | | CACTCCTCGCCCTATTGGC |
|  | Reverse | | CACTCCTCGCCCTATTGGC |
| *PPAR-γ* | Forward | | GGGATCAGCTCCGTGGATCT |
|  | Reverse | | TGCACTTTGGTACTCTTGAAGTT |
| *C/EBP-α* | Forward | | TTGATTTCTCCAGCATTTCT |
|  | Reverse | | TCCCGGGTAGTCAAAGTCAC |
| *FABP4* | Forward | | ACTGGGCCAGGAATTTGACG |
|  | Reverse | | CTCGTGGAAGTGACGCCTT |
| *SOX9* | Forward | | AGCGAACGCACATCAAGAC |
|  | Reverse | | CTGTAGGCGATCTGTTGGGG |
| *COL2A1* | Forward | | TGGACGATCAGGCGAAACC |
|  | Reverse | | GCTGCGGATGCTCTCAATCT |
| *ACAN* | Forward | | GTGCCTATCAGGACAAGGTCT |
|  | Reverse | | GATGCCTTTCACCACGACTTC |
| *ANGPT1* | Forward | | TCGTGAGAGTACGACAGACCA |
|  | Reverse | | TCTCCGACTTCATGTTTTCCAC |
| *ANGPT2* | Forward | | AACTTTCGGAAGAGCATGGAC |
|  | Reverse | | CGAGTCATCGTATTCGAGCGG |
| *CFD* | Forward | | GACACCATCGACCACGACC |
|  | Reverse | | GCCACGTCGCAGAGAGTTC |
| *IGFBP2* | Forward | | GACAATGGCGATGACCACTCA |
|  | Reverse | | CAGCTCCTTCATACCCGACTT |
| *PTX3* | Forward | | CATCTCCTTGCGATTCTGTTTTG |
|  | Reverse | | CCATTCCGAGTGCTCCTGA |

Abbreviations: *GAPDH*, glyceraldehyde-3-phosphate dehydrogenase; *ACTB*, actin beta; *U6*, small nuclear RNA U6; *MALAT1*, long noncoding RNA metastasis associated lung adenocarcinoma transcript 1; LncRNA *ROA*, Long noncoding RNA repressor of adipogenesis; *ALP*, alkaline phosphatase; *RUNX2*, Runt related transcription factor 2; *OSX*, osterix; *OCN*, osteocalcin; *PPAR-γ*, peroxisome proliferator-activated receptor gamma; *C/EBP-α*, CCAAT/enhancer binding protein alpha; *FABP4*, fatty acid binding protein 4; *SOX9*, SRY-box transcription factor 9; *COL2A1*, collagen type II alpha 1 chain; *ACAN*, aggrecan; *ANGPT1*, angiopoietin 1; *ANGPT2*, angiopoietin 2; *CFD*, complement factor D; *IGFBP2*, insulin-like growth factor binding protein 2; *PTX3*, pentraxin 3.

**Supplementary Table S4. Sequence of siRNAs for gene knockdown.**

| **siRNA** | **Sequence (5’-3’)** | |
| --- | --- | --- |
| NC | Sense | UUCUCCGAACGUGUCACGUTT |
|  | Antisense | ACGUGACACGUUCGGAGAATT |
| lncRNA *ROA* S1 | Sense | GGAAGUGAUGUAUGCACUUTT |
|  | Antisense | AAGUGCAUACAUCACUUCCTT |
| lncRNA *ROA* S2 | Sense | GCCCGACUUUGUGCAGCAATT |
|  | Antisense | UUGCUGCACAAAGUCGGGCTT |
| lncRNA *ROA* S3 | Sense | GCAUAAGCCAAGCUAACUUTT |
|  | Antisense | AAGUUAGCUUGGCUUAUGCTT |
| *IGFBP2* S1 | Sense | GGUUGCAGACAAUGGCGAUTT |
|  | Antisense | AUCGCCAUUGUCUGCAACCTT |
| *IGFBP2* S2 | Sense | ACCUCUACUCCCUGCACAUTT |
|  | Antisense | AUGUGCAGGGAGUAGAGGUTT |
| *IGFBP2* S3 | Sense | CCUGUACAACCUCAAACAGTT |
|  | Antisense | CUGUUUGAGGUUGUACAGGTT |
| *PTX3* S1 | Sense | GCACAAAGAGGAAUCCAUATT |
|  | Antisense | UAUGGAUUCCUCUUUGUGCTT |
| *PTX3* S2 | Sense | GGGAUAGUGUUCUUAGCAATT |
|  | Antisense | UUGCUAAGAACACUAUCCCTT |
| *PTX3* S3 | Sense | GGAGCUCAGUAUGUUUCAUTT |
|  | Antisense | AUGAAACAUACUGAGCUCCTT |

Abbreviations: NC, negative control; lncRNA *ROA*, long noncoding RNA repressor of adipogenesis; *IGFBP2*, insulin-like growth factor binding protein 2; *PTX3*, pentraxin 3.

**Supplementary Table S5. Information of used primary antibodies.**

| **Target** | **Source** | **Company** | **Catalog #** | **Dilution** |
| --- | --- | --- | --- | --- |
| GAPDH | Mouse | Cell Signaling Technology | #97166 | 1:2000 for WB |
| RUNX2 | Rabbit | Cell Signaling Technology | #12556 | 1:1000 for WB |
| OCN | Rabbit | Abcam | #Ab133612 | 1:1000 for WB |
| FLAG | Rabbit | Cell Signaling Technology | #2368 | 1:2000 for WB |
| PPAR-γ | Rabbit | Cell Signaling Technology | #2443 | 1:1000 for WB |
| C/EBP-α | Rabbit | Cell Signaling Technology | #2295 | 1:1000 for WB |
| FABP4 | Rabbit | Cell Signaling Technology | #2120 | 1:1000 for WB |
| SOX9 | Rabbit | Cell Signaling Technology | #82630 | 1:1000 for WB |
| IGFBP2 | Rabbit | Cell Signaling Technology | #3922 | 1:1000 for WB |
| PTX3 | Rabbit | Abcam | #Ab190838 | 1:1000 for WB |
| β-catenin | Rabbit | Cell Signaling Technology | #9582 | 1:2000 for WB |
| N-p-β-catenin | Rabbit | Cell Signaling Technology | #4270 | 1:2000 for WB |
| AKT | Mouse | Cell Signaling Technology | #2920 | 1:2000 for WB |
| pAKT | Rabbit | Cell Signaling Technology | #4060 | 1:2000 for WB |
| ERK1/2 | Rabbit | Cell Signaling Technology | #4695 | 1:2000 for WB |
| pERK1/2 | Rabbit | Cell Signaling Technology | #4370 | 1:2000 for WB |
| JNK | Rabbit | Cell Signaling Technology | #9252 | 1:2000 for WB |
| pJNK | Rabbit | Cell Signaling Technology | #9255 | 1:2000 for WB |
| hnRNP A1 | Rabbit | Cell Signaling Technology | #8443 | 1:1000 for WB  1:50 for ChIP |
| Perilipin-1 | Rabbit | Abcam | #Ab3526 | 1:100 for IHC |

Abbreviations: WB, western blot; ChIP, chromatin immunoprecipitation; IHC, immunohistochemistry.

**Supplementary Table S6. Primers Used for ChIP**

| **ChIP primers** |  | **Primer sequence (5’-3’)** | |
| --- | --- | --- | --- |
| Primer 1^*^ | Forward | | TCCTTGCCTCGAAACCTTGT |
|  | Reverse | | ACTGGGTAAACCTAGAATGGGG |
| Primer 2^**^ | Forward | | TTTCTCCCCTACCACCCCTC |
|  | Reverse | | GGAGAGCGAGGGAAATGTGG |

^*^Primer 1 targets at -1013 to -878 of the *PTX3* promoter.

^**^Primer 2 targets at -314 to -127 of the *PTX3* promoter.

**Supplementary Material. Sequence of lncRNA ROA**

LncRNA Repressor of Adipogenesis (*ROA*), 2030nt.

>lncRNA *ROA*

GATCTGGCAGGGCTTTAAAGTAATGCCAGAGGTCCTTCCAGGCAGTTCTC

AACTTGCTCCTTGGCTGTTCTCCTGACACTTAAGTCAGAGCATTCTGTAG

CTGTATTTTACAGAACTGTAATTGCACAGATTGTTCCTCTTACAAGAGAA

ATATCCAGAGCTCTAAGAAAACAACTGATGAAAACGACATGCGTGACATT

TGGTGCTGAAGACCCAGGTCAGAGAGACTCCTTTGGGGGACCAGTCCACT

GTCCTTGCCCTCACTCTGTGAGGAGACCCACCTACGACCTCGGGTCCTCA

GACCAACCAGCTCAAGGAACATCTCACCAATTTCAAATCGGGACCCCACT

GGAAAATGGACTGTCCAACTCACCCGGCAGCCACTCCCAGAGCCCCTGGA

ACTCTGGCCCAAGGCTCTCTGACTGACTCCTTCCCAGATCTTCTCGGCTC

AGCAGCTGAAGACTGACACTGCTTGATTGCCTGGGAAGCCTATAGGACCA

TCACAGACACTTTGGATAACTCTTACAGTGAAGGACAAAAAAGCGCCACT

GCAGGGAGGAATGCCAGGGTCCCATAGAGGGAGACCGACAGTGAAGTGGA

GCCTGCCATGAATTCACCAAAGGCCATTCTCCCTTTCTTCCAGAAGAACA

GCATTTAGCTGGGCCTATGGCTACCTACCTAAAGACTGCACCTCTTAGTC

ACCCTTGTGGCCAGGTGTGGCCACGTGACAGTGGAATGTAAGTGGAAGTG

ATGTATGCACTTGGGTTCATTGGTTTAAAAAAAAATATGACCGCCCTCCA

TTTCCACTCTTTGTCTTATCTGTGAATGGGAACATGGCTGTGTCTGCAGC

CCGACTTTGTGCAGCAAAGGACAAGGCCCTAGGGGAAGGTGGAGAAACAA

GATGGAACAACTTGGACCCAACAACTTGGATCCCTATGTGGCCATGTGGA

GTCATCTCACCCACCTGGGCCACTCACCCCGAAGACTGTTGTGTGAGGGT

GAAATAAACTTCTGTATCACTAGCCCCTGCATTTCAGAAATTCTTTGTTA

TGGCAGTTTTGCCTTTATCCTAACACAGACAGCAAAGTATCTTCTGTGGC

CCCATGACTAATTTCGTTGTTTAAACTCATTATAAAAACTATATAAATAA

AATATTAAAAATCAAATAAAATAGCAAGATAACACTGTGTAACTATCCAG

TTTTAGATGTTATTACCTTTTTGGCATATTTACCTCATCTATTTTCTCCA

GGGGTTTTTAAAGAAGAGTCATGCTTGTTATTTTAAATTATCTGCAGTGC

TCCTTGGTCTTATAAAACTATGGGTTATTTCAAACTTCGGTAAATGTCTG

ATACACATGAACTTCTTGCATTGTTGAGGAAATCAGAGAAAAATGATGCT

GTTTTTCCCTTGTAAGTTGACAATGGATGGAGATGAAACCACCTTTGCAA

AATTATGGCAATAAGAGAAATTGGACATGGCAGACTCCATCTTGCTTTTA

GCCTCACAGGTTGGCTGTCTTGGCTCATTCCTGGGCATAAGCCAAGCTAA

CTTTGGCAGAAATTGAGTTTATAGTGTAAACGATAATAGCCTTTCCCCAA

AACTAAACTACCCTTGTAAAATTAATGAAAGGCCACCAAGTTAGGAGGAT

GAGAAGGGCCTGAATGCCTGAATTCAGCCATTATTCTGGAAGTCACAAGA

TGTGCAACCTCCCCAGTTACTCCTGTAAATAACTTCAGTATTGTAGAACC

TAAGATTGGCCTTTTGACAGCCTTTTGCATTTCTGACAACTAGATGGCCC

CACGCAGACCCGTGACTCAACCAGTCCTATAGCCTTCATCCAAAAATGGA

CTCAGTGCATGAGGACTGTTTTCCACACCCTTATGATTGCATCCCAAACC

AATCAGCAGCACCCATACCCTATCCCCTGCACACCAAACTATCTTTGAGA

AACTCCTAACCTCTGATTCTTTGGTGAGATTGATTTAAGTAATAACTCTG

TCTCCCACATGGCGTGGCTGGCGTCGTGTC
